# Comparative Review for Enhancing CO_2_ Capture Efficiency with Mixed Amine Systems and Catalysts

**DOI:** 10.3390/molecules29194618

**Published:** 2024-09-29

**Authors:** Wenhao Jiang, Yuchen Lin, Chengqi Sun, Yin Sun, Yunlong Zhu

**Affiliations:** 1 Naval Architecture and Shipping College, Guangdong Ocean University, Zhanjiang 524088, China; 18256533425@stu.gdou.edu.cn (W.J.); linyuchen@stu.gdou.edu.cn (Y.L.); ysun@gdou.edu.cn (Y.S.); 2 Guangdong Provincial Key Laboratory of Intelligent Equipment for South China Sea Marine Ranching, Guangdong Ocean University, Zhanjiang 524088, China; 3 College of Power and Energy Engineering, Harbin Engineering University, Harbin 150001, China; 3119216735@hrbeu.edu.cn

**Keywords:** CO_2_ capture, organic amine absorption, mixed amine systems, novel amines, catalyst

## Abstract

This study investigates methods to enhance the efficiency of CO_2_ capture using organic amine absorption and compares the performance of traditional and novel amine solvents. It reviews various single-component and mixed amine absorbents, as well as catalysts used in these methods, highlighting the superiority of mixed amine absorbents over single-component amine absorbents in CO_2_ absorption and desorption. Additionally, the study explores the catalytic mechanisms and effects of catalysts in the CO_2_ absorption/desorption process with amine solvents and provides an outlook on future research directions. The aim is to promote the widespread adoption of organic amine absorption technology in industrial applications and to contribute to the development of more sustainable and efficient CO_2_ capture technologies.

## 1. Introduction

Climate change has garnered widespread global attention. The rise in global temperatures, glacier melting, and increasingly frequent extreme weather events are alerting humanity to the critical importance of addressing climate issues. Climate change is mainly caused by excessive emissions of greenhouse gases, of which carbon dioxide (CO_2_) is a major contributor. Studies indicate that even by 2040, human dependence on fossil fuels is projected to remain at approximately 74% [1]. In 2022, total global anthropogenic greenhouse gas emissions in CO_2_ equivalents reached 36.8 billion tons, with an average annual increase of 2.5% since 1950 [2]. Without effective measures, CO_2_ emissions will continue to increase, further exacerbating the threats to human living environments.

**Figure 1:**
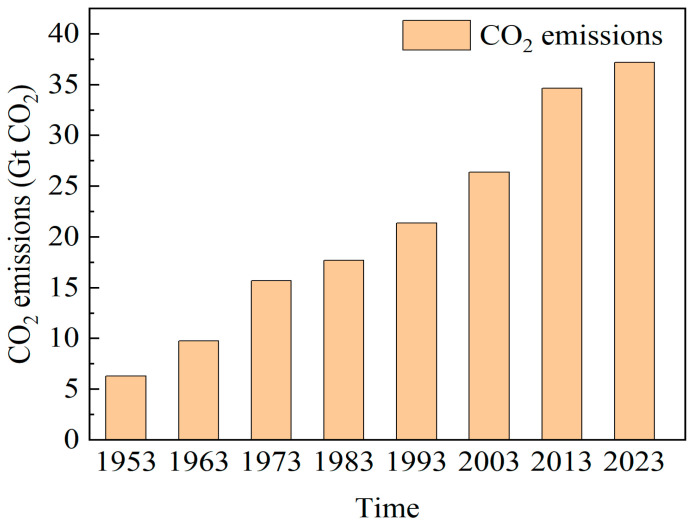
Total energy-related CO_2_ emissions 1953–2023.

To mitigate CO_2_-related environmental damage, countries signed the Paris Agreement, aiming to reduce emissions by 40% by 2030 [3]. However, global energy-related CO_2_ emissions, as reported by the International Energy Agency, continue to rise (Figure 1). In 2023, CO_2_ emissions reached 37.2 Gt [4]. This trend indicates that effective measures are urgently needed to limit global carbon emissions; otherwise, the environmental threats will become even more severe. Consequently, finding effective carbon reduction methods has become a prominent research focus.

The burning of fossil fuels in the energy sector is closely linked to increased carbon emissions. For a long time into the future, the position of fossil fuels as the primary energy source cannot be replaced. To address the climate crisis caused by excessive carbon emissions, three main approaches have generally been considered: (1) increasing efficiency on the demand side (by improving energy use) and on the supply side (by producing energy more efficiently); (2) replacing fossil fuels with clean energy; and (3) capturing CO_2_ from burning fossil fuels [5]. However, merely pursuing energy efficiency may backfire, as maintaining the current level of efficiency could slow down the growth rate of industrial production and fail to meet the increasing demand for human services [6]. To control carbon emissions at an environmentally harmless level by improving energy efficiency, it would be necessary to achieve zero fossil fuel use by 2050 and maintain the energy supply at the 2020 level throughout these 30 years. Achieving this goal would require increasing the annual growth rate of renewable energy production by 6 to 8 times, which presents a significant challenge. Additionally, despite the increase in oil prices over the past 20 years, its cost remains relatively low compared to alternative energy sources [7]. Therefore, developing low-carbon renewable energy systems to replace fossil fuels is a long-term process as fossil fuels remain the primary source of energy at present. CO_2_, which mainly originates from coal-fired power plants and various industrial emission sources, can be captured at the source using carbon capture technology applied to industrial production equipment, thereby reducing CO_2_ emissions [8]. Carbon capture technology has been used in industrial production for over 80 years, accumulating extensive practical experience. Consequently, carbon capture remains a crucial technological approach to addressing the climate crises caused by carbon emissions.

Numerous reviews have examined CO_2_ capture technologies. Significant improvements to existing CO_2_ capture processes to enhance overall CO_2_ removal efficiency were explored by Mondal et al. [9]. The regeneration energy consumption and degradation issues of amine solutions used in CO_2_ capture processes were investigated by Meng et al. [10]. The stability and reusability of bifunctional materials as adsorbents were studied by Omodolor et al. [11]. The performance of physical adsorption technologies for CO_2_ was evaluated by Liu et al. [12]. The CO_2_ capture performance and challenges of liquid absorption methods were reviewed by Ochedi et al. [13]. These CO_2_ capture methods exhibit excellent performance and have made significant contributions to advancing CO_2_ capture technology. However, these methods more or less exhibit issues, such as suboptimal CO_2_ capture efficiency and regeneration energy consumption levels that exceed the acceptable range for current industrial production. This study focuses on amine absorption, a technology with good CO_2_ capture efficiency, and explores two methods to effectively reduce regeneration energy consumption: mixed amine solvents and catalysts. Amine absorption is regarded as one of the most advanced and commercially promising technologies for post-combustion CO_2_ capture [14]. For decades, satisfactory results have been achieved in the chemical absorption of CO_2_ using amine solutions [15].

This paper investigates amine absorption, reviews the CO_2_ capture performance and reaction mechanisms of amine solvents, and outlines future development directions.

## 2. CO_2_ Capture Technology

**Figure 2:**
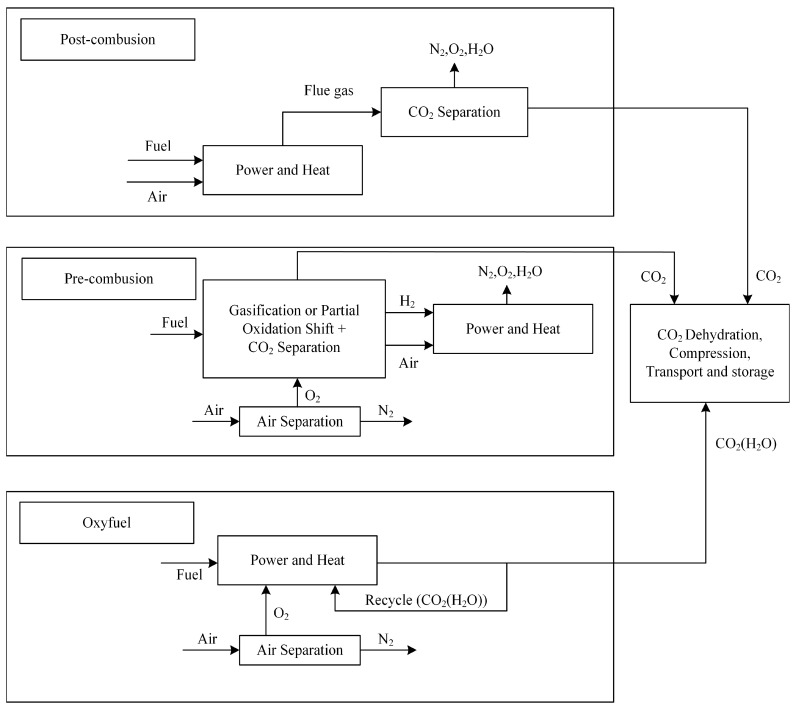
Carbon capture approaches and technology options [17].

The current suite of CO₂ capture technologies encompasses three principal approaches: pre-combustion capture, post-combustion capture, and oxy-fuel combustion [16]. Research by Wang et al. details the specific processes of these three carbon capture technologies (Figure 2) [17]. In practical applications, these technologies can be implemented at suitable locations within various industrial systems to achieve efficient CO_2_ capture.

Pre-combustion carbon capture is a technology that converts fossil fuels into syngas (H_2_ and CO) before combustion, and is primarily used in coal-fired power plants. This syngas is then employed in a subsequent reaction, known as the water–gas shift reaction, whereby it is transformed into H_2_ and CO_2_ [18]. H_2_, as a clean energy source, supplies the power required for industrial production, while the resultant CO_2_ is separated using pressure swing adsorption or membrane separation technologies. However, pre-combustion capture necessitates extensive and costly infrastructure for fuel gasification, which limits the development of this technology [19].

Oxy-fuel combustion replaces the combustion environment with an oxygen-rich atmosphere [20]. The flue gas produced in this process is primarily composed of CO_2_ and water vapor. The CO_2_ is captured through the condensation of the water vapor and the separation of other gases. This technology can significantly enhance thermal efficiency, reduce pollutant emissions, and lower fuel consumption [21]. However, separating oxygen from air before combustion and creating an entirely oxygen-rich combustion environment both necessitate additional costs and energy, as well as specialized equipment for oxygen separation, which undoubtedly raises costs and impacts overall efficiency in industrial processes.

Post-combustion carbon capture technology differs from the other two methods by treating flue gas emissions after fuel combustion. This technology requires minimal modifications to the existing industrial production infrastructure and allows for the integration of CO_2_ capture components into the current system [22]. However, unlike pre-combustion carbon capture, which provides higher CO_2_ partial pressures, post-combustion carbon capture has lower CO_2_ partial pressures (approximately 13–15% of flue gas at atmospheric pressure), which limits capture efficiency. Additionally, the presence of additional gases within the flue gas stream serves to diminish the efficacy of CO_2_ capture processes.

Overall, post-combustion CO_2_ capture is regarded as the most feasible method under current industrial conditions compared to the other two approaches. Several post-combustion carbon capture technologies are currently available, including membrane separation, cryogenic separation, amine scrubbing, and solid adsorption (both physical and chemical) [23].

Membrane separation technology efficiently extracts CO_2_ from gas mixtures using highly selective membranes. It benefits include low energy consumption, reduced costs, a compact footprint, and ease of integration into existing industrial processes [24]. However, its issues include membrane material plasticization, the need for regular maintenance, and a trade-off between selectivity and permeability impact performance, necessitating further research and development [25].

Cryogenic separation technology captures CO_2_ from flue gases and syngas through phase separation induced by refrigeration and the physical phase change of CO_2_. This method avoids chemical emissions from solvent use and reduces the need for modifications to steam power cycles and utilities [26]. However, its high energy consumption limits the technology’s applicability, necessitating further cost reductions and efficiency improvements.

Solid adsorption employs adsorbents to selectively capture CO_2_ from gas mixtures by forming chemical bonds or weak molecular interactions with CO_2_ molecules. This method has advantages, including lower CO_2_ partial pressure requirements and decreased energy consumption for regeneration [27]. However, adsorbents require further enhancement in capture performance, cycle life, and durability through repeated cycles [28,29].

**Figure 3:**
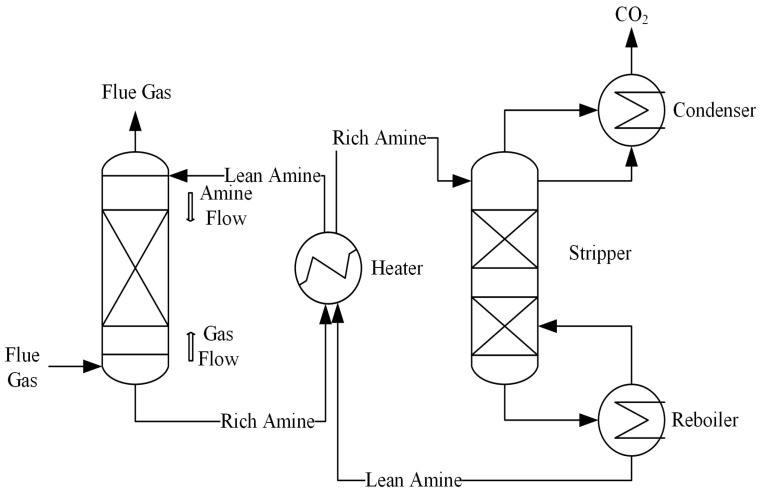
The process of organic amine absorption [30].

As shown in Figure 3, amine scrubbing (or organic amine absorption) entails the selective interaction of the solvent with CO_2_ in flue gas to produce stable compounds, thereby facilitating CO_2_ removal [30]. The solvent, after absorbing CO_2_, is referred to as the rich solution. This rich solution is then regenerated in a regeneration tower and reused for CO_2_ capture, completing a cyclic process. Amine scrubbing technology, known for its high economic efficiency, effective capture performance, and near-maturity, has been widely commercialized [31], demonstrating significant research value. In this process, the choice of amine solvent is a critical factor influencing capture process efficiency [32].

## 3. CO_2_ Capture by Organic Amine Absorption Method

Among all carbon capture technologies, absorption and adsorption are the two most widely used techniques. Among these, absorption technology is leading in carbon capture. Organic amine solutions serve as absorbents in this technology. Absorption technology involves introducing CO_2_ into a solvent, where it reacts chemically with the solvent (typically a chemical solvent) to remove CO_2_ from exhaust gases. This study primarily focuses on organic amine absorbents. The principle of amine solvent absorption involves CO_2_ reacting with the solvent to form compounds, which can then be desorbed by altering external conditions (such as pressure and temperature), allowing the absorbent to be reused.

Rectisol, developed by Linde A.G. in the 1950s, was one of the first physical solvents to be used for gas purification, primarily utilizing methanol to purify synthesis gas [33]. However, the high vapor pressure of methanol and the need for refrigerated storage make this capture method costly and of limited practical value. Subsequently, other physical solvents, such as Selexol and Purisol [34], have been introduced. Nevertheless, the efficacy of these solvents in CO_2_ capture is constrained. In fact, they typically require desorption at moderately higher temperatures and lower pressure to improve the overall efficiency of the CO_2_ capture process.

In the mid-20th century, chemical solvents, particularly amine-based ones, opened new possibilities for gas separation. Against this background, the potential of a 30 wt% solution of monoethanolamine (MEA) was identified by researchers as a promising avenue for further investigation. This chemical solvent selectively absorbs CO_2_ with high capacity and rapid absorption rates, thus being regarded as an excellent CO_2_ absorbent. MEA was subsequently widely applied in power plants and other industries. Nevertheless, a significant disadvantage of MEA absorbents is their considerable energy consumption during the regeneration process, which accounts for approximately 65% of the total energy consumption in power plants, or 3.7 GJ/t CO_2_ [35]. Nevertheless, due to its excellent absorption performance, MEA remains widely used and serves as a benchmark for evaluating new absorbents.

With industrial development, a greater variety of organic amine solvents have been developed. Although many organic amine solvents demonstrate good CO_2_ capture performance, they also have several drawbacks. The primary issues limiting the application of organic amine solvents are their corrosiveness to equipment and high energy consumption during regeneration [36]. Additionally, other components in flue gas can affect organic amine solutions. O_2_, SO_X_, NO_X_, and acidic impurities in flue gas react with organic amines, degrading the solvent’s active components [37]. To date, no chemical absorbent has managed to achieve all three key characteristics: good absorption performance, low energy consumption, and stability without susceptibility to degradation. To address this issue, researchers have focused on developing new single-component amine absorbents and improving traditional organic amine absorbents to achieve more effective and balanced CO_2_ capture characteristics. The following sections will detail technological advancements in organic amine absorbents for carbon capture and methods to enhance CO_2_ capture performance.

## 4. Amines and Catalysts for CO_2_ Absorption Technology

There are primarily two methods for CO_2_ absorption using organic amines: single-component amine absorption and mixed amine absorption. The difference between these two methods lies in the number of amine absorbents used; single-component amines use one type of amine as the absorbent, while mixed amines use a combination of two or more types of amines. There are many types of amine absorbents applicable to organic amine absorption methods. Currently, three types of amine absorbents are widely studied and used: primary amines, secondary amines, and tertiary amines [38].

The reaction mechanisms of CO_2_ absorption by primary and secondary amines are typically explained using the zwitterionic mechanism. The zwitterionic mechanism was initially proposed by Caplow [39] and further studied by Danckwerts [40]. According to the zwitterionic mechanism, the reaction between CO_2_ and primary or secondary amines yields protonated amines and carbamate esters [39]. The reaction equations involved in the process are shown in Equations (1) and (2) [41].

Initially, CO_2_ reacts with primary/secondary amines to form the R_1_R_2_NH–zwitterion:

R_1_R_2_NH + CO_2_ ↔ R_1_R_2_NH^+^COO^−^ (1)

Subsequently, deprotonation of the R_1_R_2_NH–zwitterion occurs through reaction with a base (B), forming carbamate esters. The base (B) can be primary or secondary amines (R_1_R_2_NH), OH^−^, or H_2_O:

R_1_R_2_NH^+^COO^−^ + B → R_1_R_2_NCOO^−^ + BH^+^. (2)

Due to the absence of hydrogen atoms bonded to the nitrogen atom and the signifi-cant steric hindrance caused by the alkyl groups on the nitrogen, tertiary amines cannot react directly with CO_2_ to form carbamate esters. However, tertiary amines can be reacted with CO_2_ to form bicarbonate ions and amines with protonation, thereby achieving CO_2_ absorption. Donaldson’s proposed mechanism, in which tertiary amines react with CO_2_ via a base-catalyzed hydration mechanism, explains the reaction mechanism of CO_2_ absorption by tertiary amines [42]. The absorption reaction of tertiary amines with CO_2_ is presented in Equation (3):

R_3_N + CO_2_ + H_2_O → R_3_NH^+^ + HCO_3_^−^ (3)

Research shows that primary and secondary amines have fast CO_2_ absorption rates but low capacities and high regeneration energy requirements. In contrast, tertiary amines offer high absorption capacities and low regeneration energy needs, though they absorb CO_2_ more slowly [43]. This suggests a trade-off between absorption rate, capacity and regeneration energy for amine absorbents, making it challenging for organic amine solvents to simultaneously achieve fast absorption rates, high absorption capacities, and low regeneration energy consumption. To resolve this issue, researchers are focusing on improving the capture performance of traditional organic amine solutions. Specific research directions include developing new organic amine absorbents, mixed amine solutions, and exploring catalysts [44]. Combining these methods has led to further exploration into resolving these contradictions, emerging as a new research hotspot in CO_2_ capture technology development [41].

### 4.1. Single-Component Amine Solution

Using single amine solutions to capture CO_2_ is called monocomponent amine absorption. Traditional organic amine absorbents include MEA, methyldiethanolamine (MDEA), piperazine (PZ), and 2-amino-2-methyl-1-propanol (AMP). To enhance the capture efficiency of single-component amine solutions, researchers have developed several novel amine absorbents, including 2-(2-aminoethoxy)ethanol (DGA), diethanolamine (DEA), diethylenetriamine (DETA), and 2,6-diisopropanolamine (DIPA). Studies indicate that these novel amine solutions exhibit superior CO_2_ capture performance compared to traditional ones.

Drawing on existing literature [45,46,47], this paper summarizes the CO_2_ capture characteristics of various single-component organic amine solvents and provides a detailed comparison of their performance against MEA, as presented in Table 1.

#### 4.1.1. Conventional Absorbents

Most conventional absorbents were developed in the last century and remain widely used today. However, with the growth of industrial production, the CO_2_ capture performance of these traditional organic amines is gradually failing to meet demand. Consequently, researchers are looking for ways to improve the performance of the traditional organic amines in CO_2_ capture.

##### MEA Solvent

MEA, a primary amine, is a colorless, transparent, viscous liquid at room temperature and is hygroscopic. MEA exhibits strong basicity and good chemical reactivity, reacting with CO_2_ to form carbamate compounds. These compounds can be decomposed by heating to release CO_2_, thus achieving CO_2_ removal [48]. MEA is the most widely used absorbent in CO_2_ capture research, known for its rapid absorption rate, high efficiency, and substantial CO_2_ loading capacity. However, MEA, as a chemical solvent, is prone to side reactions during CO_2_ absorption, which increases regeneration energy consumption. Additionally, the resulting carbamate compounds are chemically stable and difficult to decompose, limiting CO_2_ desorption efficiency. Decomposing carbamate compounds requires high temperatures, further increasing energy demand.

The most widely used MEA solvent is a 30wt% MEA solution, but energy consumption has become a major limiting factor in its development. It takes 3.6 to 3.8 GJ of energy to regenerate MEA to absorb one tonne of CO_2_, which, including CO_2_ compression, reduces the thermal efficiency of the power plant by 11% to 15%. The cost of regenerating MEA represents 70–80% of the total operating cost, demonstrating the need for improvement in MEA solvents [49]. During the CO_2_ absorption process with MEA, side reactions (such as interactions with other gases in flue gas and the corrosion of tower walls) lead to unnecessary MEA consumption and the formation of hard-to-degrade byproducts. This gradually reduces the capacity to absorb CO_2_ and reduces the efficiency of the MEA solution. Improving the reaction efficiency between MEA and CO_2_ and reducing regeneration energy consumption are key research directions for MEA.

To address these challenges, researchers have explored using catalysts to enhance performance. The impact of SnO_2_/ATP catalysts on CO_2_ capture technology was investigated by Tan et al. [50]. Since decomposing carbamates require regeneration heat above 120 °C, the high energy consumption required limits the use of MEA solutions for CO_2_ capture. Tan et al. investigated using 1/2-SnO_2_/ATP catalysts in a rich 5M MEA solution at 88 °C to desorb CO_2_. They found that the CO_2_ desorption rate increased by 265% and the desorption efficiency increased by 222%, while the thermal load of regenerating the MEA solution was reduced by approximately 52%. The high recyclability of the 1/2-SnO_2_/ATP catalyst was confirmed after 12 CO_2_ adsorption–desorption cycles. Guo et al. focused on enhancing CO_2_ desorption in MEA solution by using a solid acid catalyst (HZSM-5) embedded in a gas-permeable, liquid-impermeable α-Al_2_O_3_ tubular ceramic membrane to reduce the energy consumption of CO_2_ desorption [51]. They investigated how catalyst mass, temperature, pressure, liquid flow rate, and CO_2_ loading influence desorption rate and energy consumption through 60 experimental cycles. Results showed that at 363 K, the CO_2_ desorption rate with HZSM-5 was 46.2% higher than without it at 373 K. At a permeate-side pressure of 80 kPa, the CO_2_ desorption rate with HZSM-5 increased by approximately 46.5%, demonstrating the high performance and long-term stability of the solid acid catalyst combined with the porous ceramic membrane. Bhatti et al. aimed to find low-cost catalysts suitable for industrial production [52]. To reduce significant energy loss during solvent regeneration and considering the high cost of most catalysts, they synthesized a novel metal-impregnated activated carbon catalyst (Fe, Ni, Mo) and evaluated its performance in a benchmark 5M MEA solution at 86 °C. Experimental data indicated that the metal-impregnated AC catalyst significantly enhanced regeneration capacity. At 60 °C, the CO_2_ desorption rate reached 1.95 mmol/min after 10 min, an increase of 113%. Compared to non-catalyzed MEA solutions, the relative thermal load decreased by 21.2%. This inexpensive and scalable catalyst holds high practical value for advancing the industrial application of CO_2_ capture.

During the 1980s, the use of MEA solution alone as a CO_2_ capture absorbent was a primary research focus. Recent research has concentrated on minimizing the significant energy consumption of MEA regeneration while maintaining high CO_2_ loading capacities. Currently, adding catalysts to MEA solutions has emerged as a promising research avenue, offering significant potential for enhancing CO_2_ capture efficiency and reducing regeneration energy demands.

##### MDEA Solvent

MDEA, a tertiary amine, is characterized by high capacity, low energy consumption, and low causticity, establishing it as a typical CO_2_ absorbent. However, due to the low selectivity of MDEA absorbent for CO_2_, the CO_2_ capture rate of MDEA absorbent is relatively limited. PZ is commonly employed to activate the MDEA solution and improve the CO_2_ absorption rate [53]. The mixed amine absorbent MDEA + PZ will be investigated in detail in the following sections. This section introduces additional methods that enhance the absorption performance of MDEA.

**Figure 4:**
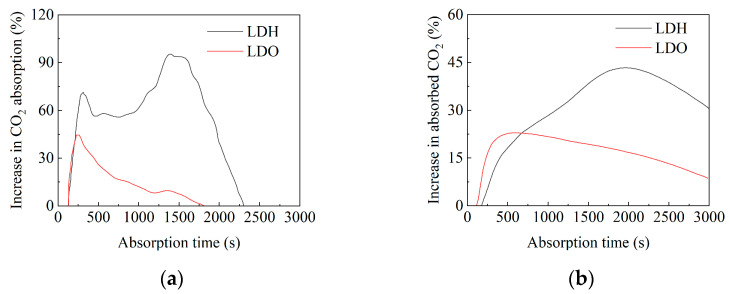
The enhancement of CO_2_ absorption process of MDEA solvent by solid base catalysts. (**a**) Percentage increase in CO_2_ absorption rate. (**b**) Percentage increase in CO_2_ absorption capacity [54].

To improve the CO_2_ absorption rate in aqueous MDEA solutions, Zhang et al. investigated five typical solid base catalysts, including MgAl layered double hydroxide (LDH) and its corresponding layered double oxide (LDO), BaCO_3_, MgCO_3_, and CaCO_3_ [54]. Experimental data presented in Figure 4 show that both LDH and LDO effectively enhance CO_2_ absorption, with LDH exhibiting significantly higher catalytic activity than LDO. Specifically, using LDH significantly increases absorption of CO_2_ compared to non-catalytic absorption. LDH-catalyzed CO_2_ absorption is a promising area of research for improving CO_2_ absorption rates in tertiary amine solutions, which will aid in the development of CO_2_ capture technologies with improved absorption kinetics, reduced energy requirements, and cost-effective solid-based catalysts. Tiwari et al. developed three laboratory-synthesized dual-functionalized ionic liquid (DFIL) promoters and evaluated their effects on enhancing the CO_2_ absorption efficiency of MDEA absorbents at high pressure conditions (2–7 bar) [55]. The DFILs, comprising 5 wt% polyamine cations (triethylenetetramine (TETA) and DETA) and cyclic amine anions (PZ and imidazole (IMZ)), were combined with 20 wt% MDEA. The physical and chemical characteristics of these mixtures were then investigated. The results revealed that the incorporation of 5 wt% DFILs into aqueous MDEA significantly improved the absorption rate along with the capacity over a range of temperatures and pressures. Notably, in the MDEA solution promoted by [TETAH][Pz], CO_2_ loading increased by 22%. The heat duty for regeneration of MDEA and mixed MDEA DFILs absorbents were found to be 25.36% and 33.34%–22.79% lower, respectively, compared to industrial MEA, with reduced absorbent lost in the regeneration process. Consequently, [TETAH][Pz] and [TETAH][Im] emerge as promising promoters for pressurized CO_2_ capture in aqueous MDEA solvent systems. Zhang et al. synthesized an efficient manganese-based oxide (MnO) catalyst using a one-step method, which significantly enhanced the CO_2_ absorption rate in MDEA solutions [56]. Experimental results showed an increase in CO_2_ absorption rate and capacity of 360% and 132%, respectively, with the MnO catalyst, surpassing most previously reported catalysts. Additionally, MnO facilitated the desorption process of MDEA and exhibited excellent recyclability. As a cost-effective and highly efficient solid catalyst, MnO effectively enhances the CO_2_ absorption ability of MDEA.

Based on the above findings, it is clear that adding activators or catalysts is an effective approach to addressing the low CO_2_ absorption rate of MDEA absorbents.

##### AMP Solvent

AMP, a single-component amine solvent, provides several advantages over MEA in specific aspects. Osagie et al. performed a techno-economic evaluation of AMP for CO_2_ capture in natural gas combined cycle (NGCC) power plants [57]. Thermodynamic assessments show that the reboiler duty in the AMP-based process is reduced by 25.6% in comparison to the MEA-based process. This reduction is primarily due to AMP solvents’ ability to regenerate at higher temperatures (140 °C) and pressures (3.5 bar), whereas MEA operates at lower temperatures (120 °C) and pressures (1.8 bar). Despite the significant reduction in efficiency losses with AMP technology for CO_2_ capture in NGCCs, the AMP-based process demonstrates superior economic performance over the MEA-based process only when the makeup rate is below 0.03%. Therefore, although AMP excels in thermodynamic performance, its economic feasibility must be thoroughly assessed when selecting a solvent.

Although AMP performs poorly as a standalone solvent in CO_2_ capture processes, it plays a crucial role as a modifier for other non-aqueous amine solvents. Non-aqueous amine solvents often cause pipeline blockages and equipment fouling due to their high viscosity and potential insoluble substances in industrial applications. To address these challenges, Ma et al. selected the polyamine 1,5-diamino-2-methylpentane (DA2MP) as the primary adsorbent and introduced AMP as a modifier to reduce solution volatility and remove substances that are not soluble in propanol (PrOH), thereby improving CO_2_ absorption performance [58]. Experimental results indicate that the AMP-modified adsorbent maintains high CO_2_ absorption capacity while significantly reducing the saturated solution viscosity to 15.00 mPa-s, much less than that of solutions without AMP modification. After four regeneration cycles, the solution retains 97% of the original CO_2_ capture capacity. Overall regeneration energy consumption is only 1.86 GJ·t^−1^ CO_2_, 50.27% less than the benchmark MEA solution, demonstrating the potential and economic benefits of AMP solvents in CO_2_ capture. Additionally, AMP serves as a modifier for solid–liquid phase change absorbents (SLPCAs) to control the physical morphology of solid products based on TETA. Research by Tu et al. indicates that AMP-modified TETA-based SLPCAs prevent gel formation and achieve easily separable crystalline powders [59]. Among the SLPCAs studied, TETA + AMP + N-methyl-2-pyrrolidone (NMP) (TETA: AMP = 2:8, with a total molar concentration of 1.0 M for TETA and AMP, and NMP as the organic solvent) exhibited the best CO_2_ capture performance, with a CO_2_ loading capacity of 0.94 mol·mol^−1^ and a regeneration efficiency of 84.14%. These studies demonstrate that AMP, as a modifier, effectively enhances the performance and regeneration efficiency of CO_2_ absorbents, highlighting its potential application value in CO_2_ capture technology.

Experimental comparisons reveal that AMP is crucial as a modifier in non-aqueous amine solutions, regulating the physical morphology of products, reducing viscosity, and preventing pipeline blockages and equipment fouling. This regulatory effect enables the application of more energy-efficient absorbents than MEA in industrial production. Therefore, AMP solvents exhibit broad potential applications.

##### PZ Solvent

PZ is the typical second-generation aqueous amine solvent used in CO_2_ capture. Compared to MEA solutions, PZ is able to operate at temperatures up to 150 °C without significant thermal degradation, reducing energy losses and minimizing the effects of degradation products [60]. PZ solvents also exhibit advantages such as resistance to oxidative degradation, low volatility, and non-corrosiveness to stainless steel. However, a challenge with PZ solutions at low CO_2_ loadings is solid phase precipitation, which needs to be addressed. To tackle the solid phase solubility issue, two main strategies can be adopted: partial substitution of PZ with 2-methylpiperazine (2MPZ) [61] and the use of semi-aqueous PZ, where PZ is mixed with water and a physical solvent (an organic compound that mixes with water but does not react with PZ). Semi-aqueous PZ is attractive because, in addition to good PZ solubility, it improves CO_2_ absorption rates [62].

PZ was identified as a highly effective promotion agent, leading to higher absorption rates in the absorption column and lower regeneration heat in the stripping tower [63]. Zhao et al. investigated the impact of adding varying amounts of water and PZ to non-aqueous amine solvents for CO_2_ capture performance [64]. Laboratory-scale tests and comparisons were conducted on energy consumption for 2-(ethylamino)ethanol (EMEA) aqueous lean amine solvent and MEA aqueous solution, with comprehensive evaluations performed in a laboratory-scale pilot plant. Experimental results indicate that with 10 wt% water content, the desorption efficiency decreases by about 10%, but adding PZ restores the solvent regeneration efficiency to 94.2%. In continuous 72-h adsorption–desorption experiments, energy consumption was reduced by approximately 45% compared to MEA aqueous solution. These results suggest that PZ aqueous solution enhances regeneration efficiency by participating in proton transfer processes. This study shows a novel and energy-efficient CO_2_ absorbent can be achieved through the combination of lean amine solvent and PZ.

The study of PZ highlights its significant application potential. Notably, PZ is non-corrosive to stainless steel, preventing damage to industrial facilities and making it more suitable for practical applications. Additionally, as a promoter, PZ can be mixed with other solvents to enhance performance. These characteristics suggest that PZ solvents excel in improving CO_2_ capture efficiency and offer high research value in reducing equipment maintenance costs and extending equipment lifespan. Therefore, PZ solvents have promising prospects for future industrial applications.

#### 4.1.2. Novel Absorbents

Traditional organic amines, such as PZ and AMP, have shown excellent performance and broad application prospects in CO_2_ capture. However, with technological advancements, researchers are increasingly focusing on novel organic amines due to their greater potential for CO_2_ capture under specific conditions. The following sections will introduce several novel organic amines and their applications in CO_2_ capture, exploring their potential to improve capture efficiency, reduce energy consumption, and adapt to various industrial environments.

##### DGA Solvent

DGA and MEA are both primary amine solvents. However, DGA is less costly than MEA, and as DGA concentration increases in the absorption solution, its absorption rate and freezing point both decrease [65]. However, the CO_2_ capture performance of DGA is not superior to that of MEA under most conditions; it performs better under specific circumstances. Salkuyeh et al. developed improvement schemes for DGA and MEA solvents at varying CO_2_ concentrations within the feed gas for reducing the energy consumption of capture devices [66]. They compared the CO_2_ capture performance using DGA and MEA at different conditions. The findings indicated that DGA is a better choice at low CO_2_ concentrations. Additionally, under low CO_2_ load, increasing CO_2_ concentration in the feed stream significantly reduces the reboiler load required for DGA, which is lower than that required for MEA. However, under high CO_2_ load conditions, the reboiler load and solvent mass flow rate required for DGA exceed those needed for MEA.

**Figure 5:**
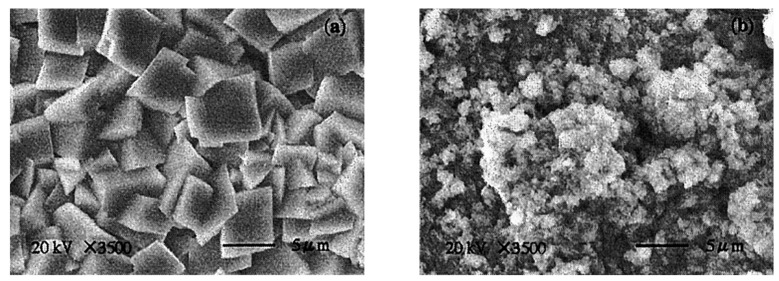
SEM micrographs of carbon steel (SB 42) after immersion in alkanolamine solutions. (**a**): MDEA; (**b**): DGA [67].

Despite DGA’s good performance in CO_2_ reactivity, its corrosion issues on capture equipment are significant. Guo et al. investigated the resistance of carbon steel to corrosion in DGA and MDEA aqueous solutions [67]. Figure 5 shows that DGA and MDEA produce different corrosive products on carbon steel. The crystalline FeCO_3_ film formed by MDEA provides effective protection against corrosion reactions on carbon steel, while the FeCO_3_ and FeC_3_ films formed by DGA do not offer similar protective capabilities. Therefore, the formation of the MDEA film inhibits both cathodic and anodic reactions, reducing corrosion on carbon steel. This highlights the potential corrosion issues associated with using DGA.

In summary, DGA, as a new absorbent, offers advantages such as low cost and excellent CO_2_ selectivity. However, its corrosion issues with equipment presents a significant limitation for its application. Therefore, future research should emphasize improving the corrosion resistance of DGA solvents to maximize their effectiveness in CO_2_ capture applications.

##### DEA Solvent

DEA, a secondary amine, exhibits high activity, low solvent cost, and better thermal stability [68]. Compared to MEA, DEA exhibits less heat on reaction with CO_2_ and produces less corrosive reaction products, but its reaction kinetics are slower [69]. Nevertheless, DEA, as a new solvent, has been extensively researched and applied in various novel processes. Kim et al. investigated a non-aqueous amine solution dissolved in alcohol, focusing on the absorption of CO_2_ by DEA in alcohol [70]. Experimental results demonstrate that high amine concentrations in non-aqueous DEA solutions can carry substantial CO_2_ loading. The absorbed CO_2_ is found within the alcohol phase, while amine and carbamate remain in the lower phase. Spontaneous phase separation of the CO_2_-loaded adsorbent allows the CO_2_-rich lower phase to be transported directly for regeneration, reducing energy consumption. These characteristics suggest that DEA–alcohol mixtures hold significant potential as CO_2_ capture absorbents. Additionally, Mavroudi et al. utilized DEA as an absorbent in membrane-based gas–liquid contactors [71]. The study results indicate that DEA enhances CO_2_ mass transfer, improves the washing capacity of liquid absorbents, and achieves up to a 99% CO_2_ removal rate, demonstrating DEA’s suitability as an absorbent in membrane contact processes.

DEA has shown a high performance in non-aqueous solutions and membrane-based gas–liquid contactors; however, its CO_2_ absorption rate is relatively slow. Currently, no effective solutions exist for this issue. Future research should concentrate on improving the absorption kinetics of DEA to enhance its effectiveness in CO_2_ capture applications.

##### DIPA Solvent

The regeneration of MEA requires high steam pressure, and due to potential evaporative losses, it is not suitable for capture processes in low-pressure environments. In contrast, DIPA can be regenerated at lower steam pressures compared to MEA, while maintaining high absorption/desorption efficiency, making it a reliable alternative absorbent [72]. To compare the capture characteristics of DIPA with other organic amine solvents, Xu et al. used a rapid screening experimental system to test and analyze the adsorption and desorption properties of five amine solutions (including N-ethylmethallylamine (EMAA), triethylamine (TEA), DIPA, N-ethyl-n-butylamine (EBA), and diallylamine (DAA)) at low critical solution temperatures, relative to 5 M MEA [73]. The study results show that 2 M DIPA performed the best, exhibiting high reaction rates under both lean and rich loadings, with a single-cycle removal efficiency of approximately 83%. Additionally, approximately 1.22 mol of CO_2_ can be removed per liter of solution per cycle, with a cycle loading of 0.611 mol CO_2_ per mol of solution. These findings highlight DIPA as a promising CO_2_ capture absorbent, which is especially advantageous in low-pressure environments, meaning it is likely to see wider adoption in industrial applications.

##### AEEA Solvent

AEEA is a novel amine solvent. As a diamine, it features both primary and secondary amines, with its molecular structure including two active nitrogen atoms and a hydroxyl (-OH) group that enhance its solubility in water. Additionally, AEEA offers a cost advantage over other novel amine solvents, making it widely used in industrial production, including the manufacture of lubricant additives, chelating agents, fuel additives, fabric softeners, and surfactants [74]. Aso et al. conducted a study on CO_2_ absorption by primary amines (AEEAp) and secondary amines (AEEAs) in AEEA and compared the CO_2_ absorption performance with that of MEA solvents [75]. The experimental results indicate that AEEAp has superior CO_2_ absorption capacity compared to MEA solvents, while AEEAs exhibits better CO_2_ desorption performance than MEA solvents. Therefore, as an absorbent containing both primary and secondary amines, AEEA solvents outperform MEA solvents in both CO_2_ absorption and desorption. This indicates its potential to replace MEA solvents as a new absorbent. Ma’mun et al. measured the gas–liquid equilibrium data for CO_2_ in a 30% AEEA aqueous solution [47]. The experimental results indicate that in CO_2_ capture processes, AEEA absorbents exhibit higher CO_2_ absorption rates and net circulation capacity compared to MEA absorbents, and maintain their CO_2_ absorption rates under higher CO_2_ loading, demonstrating more stable CO_2_ absorption performance than MEA absorbents.

Given the high absorption and desorption efficiency, low preparation cost, and stable absorption performance of AEEA, researchers widely consider it to have the potential to replace traditional absorbents as a novel absorbent.

##### DETA Solvent

DETA is a novel amine absorbent with three functional groups. The application of DETA absorbent can be divided into two aspects: as a primary absorbent and as a catalyst for other absorbents. Zhang et al. conducted CO_2_ desorption experiments using DETA solvent as the primary absorbent, comparing the CO_2_ desorption performance of DETA absorbent with MEA absorbent using regeneration heat duty as an indicator [76]. The experimental results indicate that at a temperature of 298.1 K, the kinetic rate constant of DETA is approximately 10 times that of MEA. Compared to other traditional amine solvents, DETA exhibits higher CO_2_ solubility and shows higher CO_2_ desorption rates and CO_2_ absorption capacities. When DETA is used as a promoter for other solvents, it significantly enhances the CO_2_ absorption rate. Ramezani et al. conducted CO_2_ absorption experiments using DETA solvent as a catalyst, investigating the characterization and reaction kinetics of CO_2_ absorption enhancement by DETA in K_2_CO_3_ solution [77]. The experimental results show that adding a small amount of DETA to K_2_CO_3_ solution significantly increases the CO_2_ absorption rate. Compared to solvents such as MEA, ethylaminoethanol (EAE), proline, arginine, taurine, histidine, and alanine, the CO_2_ absorption rate with added DETA in K_2_CO_3_ is the highest, demonstrating excellent catalytic performance and indicating its potential as an efficient promoter.

Based on the above findings, it is clear that whether DETA is used as an absorbent or a promoter for other solvents, it exhibits excellent CO_2_ absorption/desorption performance and promotion effects. Therefore, DETA is a highly promising novel single-component organic amine absorbent with a broad potential application range, meaning its expected to be widely used in industrial applications.

### 4.2. Mixed-Component Amine Solution

The concept of mixed amines was first proposed in 1985 by Chakravarty et al. [78]. To improve absorption efficiency and regeneration efficiency, the method of blending various amine types—primary, secondary, tertiary, and sterically hindered—into multi-component absorbents has been adopted. This approach enables the absorbent to achieve faster absorption rates, higher CO_2_ loading, and reduced regeneration energy consumption, effectively addressing the limitations of single amines. Mixed amine systems are recognized as promising absorption systems. Common mixing methods include combinations, such as primary + tertiary amines, primary + secondary amines, and primary + sterically hindered amines. Polyamines, including ethylenediamine (EDA), DETA, and PZ, are employed in mixtures to improve reaction rates and absorption capacity [79]. In summary, combining two or more different types of amines can integrate their advantages and overcome the limitations of single amines, thereby enhancing CO_2_ absorption performance.

#### 4.2.1. PZ-Based Mixed Solution

PZ can be mixed with MDEA to form an MDEA + PZ mixed amine absorbent, which exhibits good CO_2_ capture performance. Khan et al. investigated the CO_2_ absorption and desorption performance of MDEA + PZ absorbents with different ratios [80]. During the experiments, the mass percentage of PZ in the MDEA + PZ mixed amine solvent was gradually increased from 2 wt% to 10 wt%, while keeping the total solvent concentration in the aqueous solution constant at 30 wt%. The experimental results showed that as the mass percentage of PZ increased, the CO_2_ absorption rate also gradually improved. When the PZ content was at its highest (10 wt%), the absorption rate of the MDEA + PZ aqueous solution was the highest at 30.16 × 10^−6^ kmol m^−2^ s^−1^, with a maximum CO_2_ loading of 0.78 mol CO_2_. When the PZ content was at its lowest (2 wt%), the MDEA + PZ aqueous solution exhibited the highest regeneration efficiency, reaching 92.24%. Analysis of the experimental data indicates that the MDEA + PZ absorbent has good CO_2_ loading capacity and absorbent regeneration efficiency. Hosseini-Ardali et al. explored methods to improve CO_2_ capture efficiency and reduce CO_2_ capture energy consumption in flue gases using evolutionary algorithms and multi-objective optimization approaches, with different concentrations of PZ + MDEA solvents as the study subjects [81]. The experimental results showed that when the CO_2_ removal efficiency of the MDEA + PZ absorbent was 94%, the energy consumption was 2.76 GJ/t CO_2_. In contrast, in Oh et al.’s study, when the CO_2_ removal efficiency of the MEA absorbent was 90%, the energy consumption was 3.57 GJ/t CO_2_ [82]. Comparison shows that using the MDEA + PZ absorbent allows for maintaining a high CO_2_ removal efficiency while achieving lower energy consumption, making it an excellent CO_2_ capture solvent.

PZ can not only be mixed with MDEA but also with N, N, N′, N′-Tetramethyl-1,3-butanediamine (TMBPA) to form a mixed amine absorbent, which exhibits excellent CO_2_ capture performance. Aronu et al. conducted absorption and desorption experiments on different concentrations of TMBPA + PZ mixed amine absorbents at atmospheric pressure and compared the results with those of MEA at various concentrations [83]. The experimental results indicated that the 1.5 M TMBPA + 1.0 M PZ mixed amine solution had the best overall performance. This solution exhibited a high CO_2_ absorption rate and a large CO_2_ loading capacity, reaching 1.231 mol CO_2_/mol amine and absorbing 3.076 mol CO_2_ per liter of solution. Under the test conditions, the CO_2_ desorption rate of the TMBPA + PZ mixed amine absorbent was 74%, demonstrating its excellent CO_2_ carrying capacity (2.277 mol CO_2_/L). Compared to 5 M MEA, the CO_2_ cyclic capacity of the TMBPA + PZ mixed amine absorbent increased by 70% (mol CO_2_/mol amine), demonstrating significant stability. Given its excellent CO_2_ capture performance, this new mixed amine solvent holds promise for achieving higher CO_2_ capture efficiency at lower costs and has the potential to replace traditional solvents in practical industrial applications.

Based on the above findings, both PZ + MDEA and PZ + TMBPA mixed amine absorbents exhibit superior CO_2_ capture performance compared to the traditional absorbent MEA. Therefore, PZ-based mixed amine absorbents represent a promising research direction for organic amine absorbents.

#### 4.2.2. AMP-Based Mixed Solution

**Figure 6:**
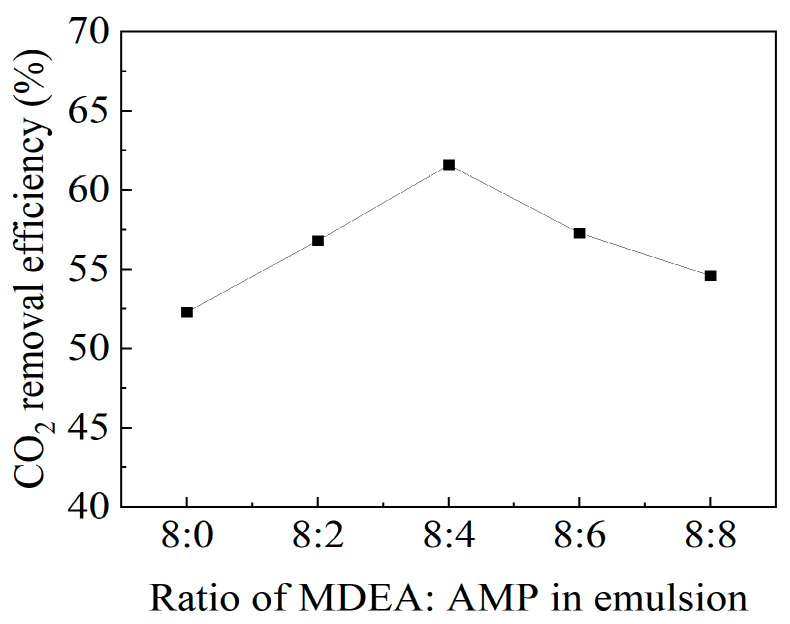
Emulsion properties under different MDEA + AMP ratio [85].

AMP absorbents have the advantage of high CO_2_ absorption capacity, but their CO_2_ absorption rate is slow. To enhance the CO_2_ absorption rate of AMP absorbents, researchers developed an MEA + AMP mixed amine absorbent, leveraging the rapid CO_2_ absorption rate of MEA to improve the CO_2_ capture performance of AMP-based mixed amine absorbents. Choi et al. investigated the CO_2_ capture performance of MEA + AMP absorbents with different ratios [84]. The experimental results indicate that adding MEA to AMP significantly improves the CO_2_ absorption rate. The CO_2_ absorption amount of the MEA + AMP mixed amine absorbent increased by 51.2% compared to a 30 wt% MEA absorbent, demonstrating that the MEA + AMP absorbent has the characteristics of high CO_2_ absorption capacity and fast absorption rate. AMP can not only be mixed with MEA to enhance CO_2_ capture performance, but can also be mixed with MDEA to form a mixed amine absorbent for CO_2_ capture. The MDEA + AMP mixed amine absorbent is commonly used in emulsion liquid membrane (ELM) technology to achieve effective CO_2_ capture. Najib et al. studied a stable ELM formulation that enhances CO_2_ absorption capacity by adding an MDEA + AMP mixed amine solvent to a NaOH solution [85]. This formulation uses an aqueous solution as the dispersed phase, kerosene as the continuous phase, and Span-80 as the surfactant to form a water-in-oil emulsion. The experimental results indicate that adding AMP to the MDEA solvent effectively improves the CO_2_ removal rate compared to using MDEA alone. As shown in Figure 6, the CO_2_ removal rate of MDEA solution without AMP is the lowest, while the highest CO_2_ removal rate of 61.6% is achieved with an MDEA/AMP ratio of 8:4. This study reveals the potential of MDEA + AMP absorbents in ELM applications. Liu et al. further investigated AMP-based mixed amine absorbents and developed a mixed amine absorbent composed of three organic amines, namely MEA + MDEA + AMP [86]. They added MDEA to the MEA + AMP mixed solution and combined the three organic amine solutions to obtain the MEA + MDEA + AMP mixed amine absorbent. The experimental results indicate that, compared to MEA absorbents, the MEA + MDEA + AMP mixed amine absorbent has a faster CO_2_ desorption rate and lower regeneration energy consumption, showing better regeneration performance.

Based on the above research findings, AMP-based mixed amine solvents have been extensively studied and can be combined with various amines to form mixed amine absorbents with enhanced CO_2_ absorption performance. AMP-based mixed solutions hold considerable research value and application potential.

#### 4.2.3. DMBA-Based Mixed Solution

Significant energy consumption for regeneration of CO_2_ limits the widespread use of amine absorption methods in industrial processes. To reduce capture costs, researchers are focusing on a more cost-effective alternative: biphasic absorbents. These absorbents exhibit phase change behavior during CO_2_ absorption. Specifically, during the absorption of CO_2_ or temperature changes, the solvent undergoes a liquid–liquid or liquid–solid phase transition, concentrating CO_2_ into a single phase (the CO_2_-rich phase), where over 90% of the CO_2_ can be captured [87]. Phase change absorbents effectively reduce regeneration energy consumption by partitioning the CO_2_-containing solvent into CO_2_-rich and CO_2_-lean phases [88]. In the absorption column, the CO_2_- lean phase persists in capturing CO_2_; meanwhile, the CO_2_-rich phase is directed to the regeneration column for CO_2_ desorption [89]. The approach in question significantly reduces the heat duty, the size of the stripping column, and CO_2_ compression workload, thus lowering regeneration energy consumption and costs. This is due to the fact that only the CO_2_-rich phase requires processing in the stripping column for regeneration [90].

**Figure 7:**
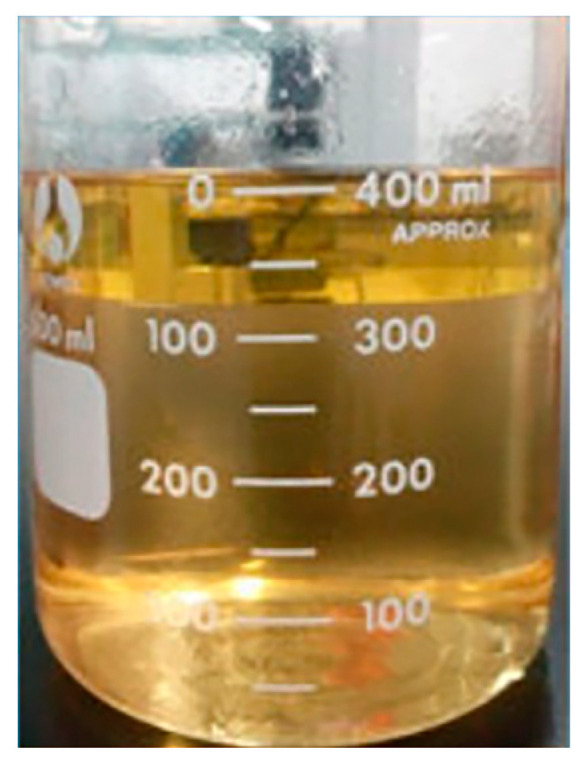
Phase transition phenomena of DMBA + DEEA mixed amine solvent [92].

**Figure 8:**
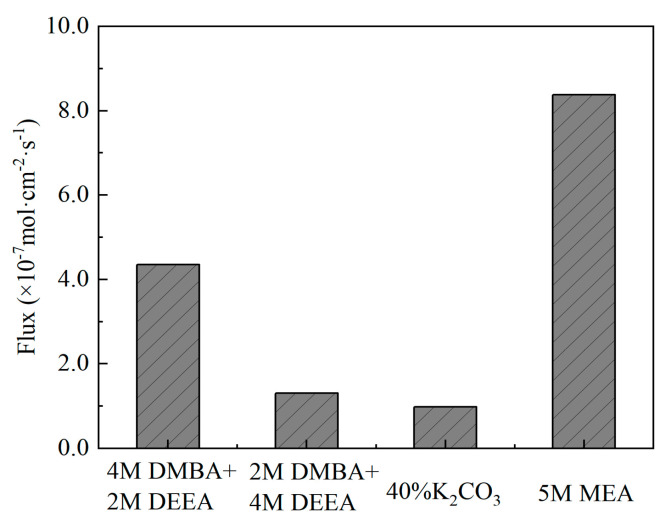
CO_2_ absorption rates of the four absorbents [92].

Biphasic absorbents are regarded as promising alternatives to MEA, with potential for significantly reducing equipment investment costs and energy consumption during the regeneration process [91]. Typically, biphasic solvents consist of primary/secondary amines as CO_2_ absorption promoters and tertiary amines as CO_2_ precipitating agents, combined in specific proportions. This section presents an organic amine mixed solvent with favorable phase change properties: N,N-dimethylbutylamine (DMBA)-based mixed amine solvent. Wang et al. investigated the blending of DMBA and N,N-diethylethanolamine (DEEA) into mixed amine absorbents to simulate CO_2_ absorption from coal-fired flue gas [92]. They prepared two different ratios of mixed amine solvents: 4M DMBA + 2M DEEA and 2M DMBA + 4M DEEA, and compared their CO_2_ absorption capacities with MEA and K_2_CO_3_. DMBA + DEEA mixed amine solvents undergo liquid–liquid separation after absorbing sufficient CO_2_, with the CO_2_ loading in the lower liquid layer reaching 2.60 mol·L^−1^ and accounting for 85% of the total solution volume (Figure 7). As shown in Figure 8, at a CO_2_ loading of 1.16 mol·L^−1^, the DMBA+DEEA adsorbent demonstrated effective CO_2_ absorption properties. Specifically, the absorption rate of 4M DMBA + 2M DEEA is 2.35 times higher than that of 2M DMBA + 4M DEEA, demonstrating superior absorption efficiency, reaction stability, and potential for further development.

Based on the above research findings, the DMBA + DEEA mixed amine solvent exhibits excellent phase change properties and high CO_2_ absorption capability, making it an ideal alternative to MEA. These solvents can significantly reduce equipment investment costs and energy consumption.

#### 4.2.4. DEEA-Based Mixed Solution

**Figure 9:**
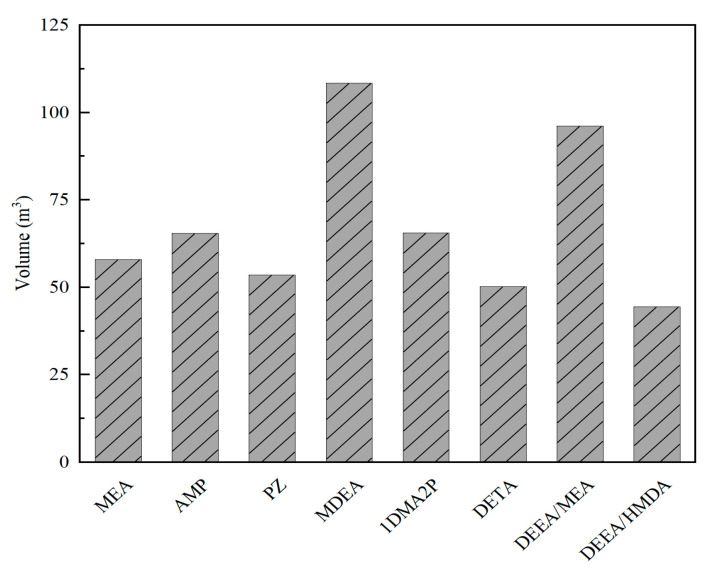
Column volumes of the eight solvents [96].

In comparison to single-component organic amine solvents, mixed amine solutions have been demonstrated to enhance the performance of CO_2_ capture while simultaneously lowering costs by reducing the volume of absorption columns required. 1,6-hexanediamine (HDMA), a primary amine with two amino groups, forms alkaline solutions that react rapidly with CO_2_, effectively separating it from flue gas. Its CO_2_ absorption capacity is approximately twice that of the MEA solvent [93]. Nevertheless, the carbamate formed as a result of the reaction between HDMA and CO_2_ is relatively stable, thereby necessitating a greater expenditure of energy during the desorption process. In contrast, DEEA, a tertiary amine, exhibits lower regeneration energy consumption but has a slower reaction rate with CO_2_. The combination of DEEA and HDMA yields DEEA + HDMA mixed amine solvents, which integrate the advantages of low desorption energy consumption and rapid CO_2_ reaction rate, positioning them as promising CO_2_ capture solvents [94]. Nevertheless, comprehensive technical analysis and validation are required to confirm the applicability of DEEA + HDMA mixed amine solvents in industrial settings [95]. Bai et al. conducted a comprehensive technical evaluation to investigate the CO_2_ capture performance of DEEA + HDMA mixed solutions and demonstrated their advantages over traditional absorbents [96]. The study reveals that the DEEA + HDMA solvent exhibits higher CO_2_ solubility (0.94 mol/mol), volumetric overall mass transfer coefficient (0.7560 kmol/(m^2^·h·kPa)), and a lower heat of absorption (67.1 kJ/mol). As shown in Figure 9, the packed column volume of DEEA + HDMA solvent is diminished by 43.5% in comparison to MEA, thereby underscoring its potential to reduce equipment costs. In conclusion, research findings validate the feasibility of DEEA + HDMA mixed amine solvents for industrial applications, showcasing their promising development potential and excellent CO_2_ capture characteristics.

The DEEA + AEEA mixed solution, as a dual-phase amine capture solvent, demonstrates superior CO_2_ absorption performance, significantly outperforming the use of DEEA alone. Kierzkowska-Pawlak et al. investigated CO_2_ absorption in DEEA aqueous solutions activated by AEEA at 303 K [97]. Three solvent formulations were tested: 2 M DEEA + 0.1 M AEEA, 2 M DEEA + 0.2 M AEEA, and 2 M DEEA + 0.3 M AEEA. The results reveal that AEEA significantly enhances CO_2_ absorption in DEEA aqueous solutions, with enhancement factors being at least double those of pure DEEA, indicating that AEEA effectively improves the absorption performance of DEEA. Additionally, mixed amine absorbents of DEEA and MEA exhibit excellent CO_2_ capture performance. Luo et al. employed a multi-rapid screening method to evaluate different MEA + DEEA ratios at a total molar concentration of 5 mol/L [98]. The findings demonstrate that the MEA + DEEA mixed system with a molar ratio of 2.5:2.5 exhibits the highest cyclic capacity, achieving 1.18 mol CO_2_/L solution. This represents a 31.8% increase in CO_2_ removal rate in comparison to the 30 wt% MEA solution. This demonstrates the superior performance of MEA + DEEA mixed amine solvents in comparison to single-component MEA solvents.

Analysis of the above research findings indicates that DEEA-based mixed amine solvents outperform single-component organic amine solvents in both CO_2_ capture performance and cost-effectiveness. This validates the effectiveness and application potential of DEEA-based mixed amine solvents as CO_2_ absorbents.

This chapter concludes that mixed amine solutions, prepared by varying solvent proportions, show significant differences in CO_2_ capture performance. Studies reveal that these mixed amine solutions generally provide superior CO_2_ absorption capabilities and higher capture efficiency than single-component amine solvents. This suggests that the synergistic effects of mixed solvents effectively enhance CO_2_ capture performance, highlighting their greater potential and advantages in practical applications.

### 4.3. Solid Catalysts for Amine Based Solution

The aforementioned research has revealed that a significant disadvantage associated with organic amine absorption is the considerable energy expenditure incurred during the desorption process. To address this issue, researchers have developed catalysts to further reduce desorption energy consumption. These catalysts reduce desorption energy consumption by overcoming the limitations of CO_2_ absorption in amine reactions. Typically, amines that absorb CO_2_ more slowly require less energy during regeneration. The use of catalysts accelerates the CO_2_ absorption process, which reduces solvent flow rates and tower size while maintaining energy efficiency during regeneration [99].

Recent studies have concentrated on improving the efficacy of solid catalysts, including solid acid and solid base catalysts, each with distinct catalytic mechanisms. Alivand et al. studied solid acid-base catalysts in detail, finding that their introduction into amine aqueous solutions significantly improves reaction pathways, enhances CO_2_ desorption efficiency during low-temperature regeneration, and reduces energy consumption [100].

Solid acid catalysts commonly used in practical applications include HZSM-5 and γ-Al_2_O_3_. HZSM-5 functions as a Brønsted solid acid catalyst by donating protons to decompose formamides, while γ-Al_2_O_3_ acts as a Lewis acid catalyst by acting as an electron acceptor to form bicarbonates. Zhang et al. studied the catalytic mechanism of Brønsted solid acid catalysts in the decomposition of formamides [101], with specific reaction processes involving Equations (4) and (5):

MEACOO^−^ + BH^+^ ↔ MEACOOH + B (4)

MEAH^+^ + B ↔ MEA + BH^+^ (5)

Lewis acids are typically formed by unsaturated metal atoms, which accept a pair of electrons. Ali Saleh Bairq et al. investigated the catalytic mechanism of Lewis acids in amine deprotonation reactions [102], with the specific reaction processes illustrated in Equations (6) and (7):

MEAH^+^ + L ↔ MEA + LH (6)

LH + H_2_O ↔ L^−^ + H_3_O^+^ (7)

**Figure 10:**
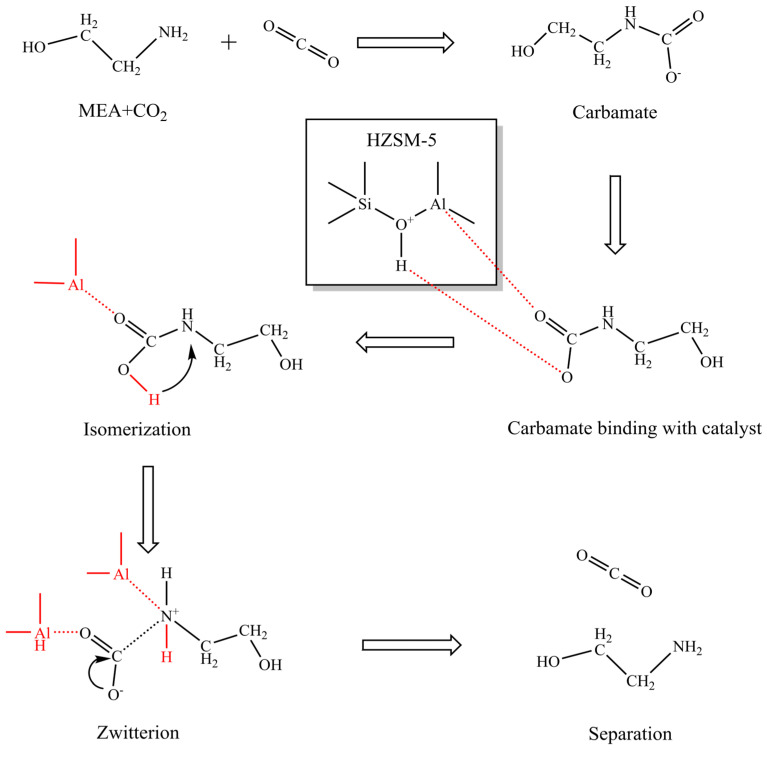
Catalytic mechanism of HZSM-5 catalyst in promoting the decomposition of carbamate during the regeneration of organic amine absorbents [104].

Shi et al. investigated the effect of HZSM-5 and γ-Al_2_O_3_ on reducing the relative thermal load during the regeneration of CO_2_-saturated MEA solvents [103]. The experimental results showed that, under the condition of 90 °C, HZSM-5 reduced the relative thermal load to 62.7%, while γ-Al_2_O_3_ reduced it to 72.5%. This indicates that HZSM-5 is more effective than γ-Al_2_O_3_ in reducing the thermal load during the MEA desorption process, thereby lowering the regeneration energy consumption at a higher rate. Analysis of the experimental data reveals that γ-Al_2_O_3_ and HZSM-5 exhibit distinct accessible acidic sites characteristics. γ-Al_2_O_3_, as a Lewis acid catalyst, has a smaller surface area, which restricts CO_2_ from accessing the active acidic sites. In contrast, HZSM-5, as a Brønsted acid catalyst, has a larger surface area and a greater number of accessible acidic sites, significantly enhancing the catalytic performance. Research results indicate that Brønsted acid catalysts, such as HZSM-5, have significant advantages in amine solutions with high CO_2_ concentrations, particularly in promoting CO_2_ desorption. As most CO_2_ is primarily released from solutions with high CO_2_ concentrations, Brønsted acid catalysts, such as HZSM-5, are preferred for promoting CO_2_ desorption. The specific catalytic mechanisms are shown in Figure 10 (The red markings indicate the attachment sites on the carbamate for both the proton (H) and the metal atom (Al)) [104].

**Figure 11:**
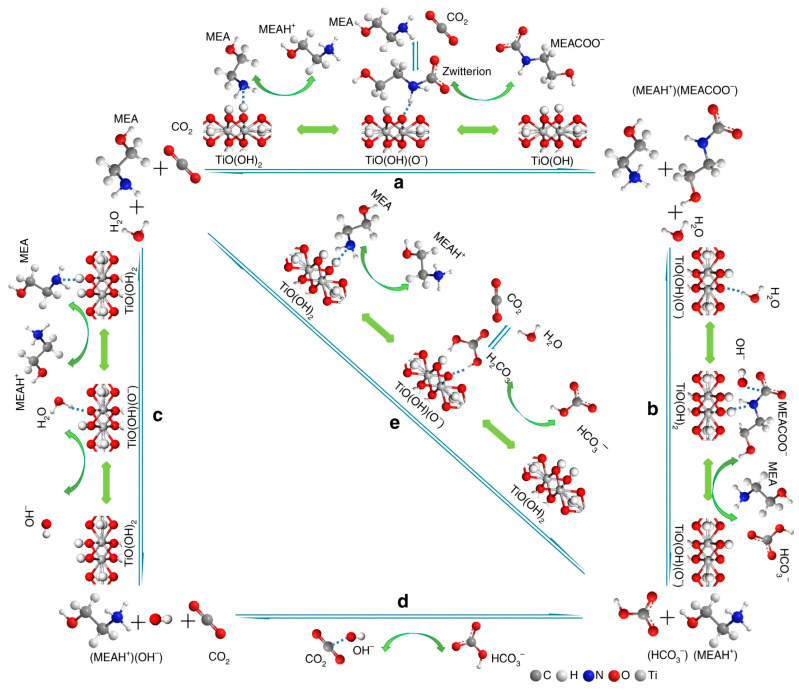
Three catalytic mechanisms of TiO(OH)_2_ catalyst in the CO_2_ absorption and desorption processes of MEA absorbent (a: The formation of carbamate (MEACOO^−^); b: The formation of bicarbonate; c: The forward reaction forming (MEAH^+^)(OH^−^); d: The reverse reaction leads to the formation of (MEAH^+^)(OH^−^); e: The formation and dissociation of carbonic acid.) [105].

TiO(OH)_2_, a basic solid catalyst, has been the subject of extensive study with a view to its potential use in the capture of CO_2_. It significantly alters reaction pathways and fundamentally reduces energy consumption. The TiO(OH)_2_ catalyst is advantageous due to its high efficiency in accelerating CO_2_ desorption, stability, and cost-effectiveness. Lai et al. investigated the catalytic mechanism of solid base catalysts [105]. The study found that the hydroxyl groups of TiO(OH)_2_ effectively donate and accept protons, greatly accelerating proton-involved reactions, especially protonation and deprotonation. As shown in Figure 11, bicarbonate can be formed through three pathways during the MEA CO_2_ absorption/desorption process, with TiO(OH)_2_ playing a promoting role in each. The first pathway involves the formation of carbamate (MEACOO^−^) via a zwitterionic intermediate, which subsequently hydrolyzes to form bicarbonate (steps a and b). The second pathway entails the forward and reverse formation of (MEAH^+^)(OH^−^), which leads to the production of bicarbonate (steps c and d). The third pathway forms bicarbonate through the binding and dissociation of carbonic acid (step e). In the first pathway, TiO(OH)_2_ donates protons to MEA and accepts protons from the zwitterion, facilitating the formation of MEAH^+^ and MEACOO^−^ during CO_2_ absorption. Additionally, it donates protons to MEACOO^−^ for its decomposition and accepts protons from the deprotonation of MEAH^+^, promoting CO_2_ desorption. In the second and third pathways, TiO(OH)_2_ assists in the deprotonation of MEAH^+^ and donates protons to HCO_3_^−^, bypassing the challenge of direct proton transfer from MEAH^+^ to HCO_3_^−^. Notably, in the reverse process of step e, TiO(OH)_2_ donates protons to HCO_3_^−^ to form carbonate, facilitating its direct decomposition to release CO_2_, thereby eliminating the need for additional energy to decompose bicarbonate. Lai et al.’s experimental results indicate that the effective absorption time for CO_2_ is extended by 66% with the addition of TiO(OH)_2_ as a catalyst compared to the absence of a catalyst. Under the same effective adsorption time, CO_2_ absorption amounts are 162 mmol without TiO(OH)_2_ and 283 mmol with TiO(OH)_2_, indicating a 75% increase. These results demonstrate the significant catalytic function of TiO(OH)_2_ in CO_2_ capture.

In conclusion, the introduction of catalysts has been demonstrated to markedly enhance the CO_2_ absorption performance of organic amine absorbents. This improvement not only increases CO_2_ capture efficiency but also significantly reduces energy consumption during absorbent regeneration. Catalysts offer an effective approach to optimizing organic amine absorption, expected to yield significant economic and environmental benefits, and promote the development and deployment of CO_2_ capture technology.

## 5. Development Trend of Amine Absorption

In the preceding sections, various types of amine absorbents and their respective advantages and disadvantages have been detailed. Researchers work continuously to improve the organic amine absorption method to enhance CO_2_ capture performance. As research progresses, the application forms of traditional organic amines evolve. Table 2 summarizes the latest applications of these traditional organic amines in CO_2_ capture.

Currently, the industrial application of organic amine absorption faces inherent challenges, particularly the high regeneration energy consumption of the absorbents. Regenerating the absorbent involves heating a large volume of water to the regeneration temperature, and the process of regenerating the absorbent after CO_2_ saturation requires a substantial amount of energy—approximately 30% of a power plant’s output [106]. Consequently, researchers are focused on developing new and effective methods to reduce the regeneration energy consumption of absorbents. Table 2 illustrates the current research hotspots in organic amine absorption methods. As shown in Table 2, mixed amine solutions and catalysts play a crucial role in improving CO_2_ capture performance and reducing regeneration energy consumption. Future research will concentrate on developing catalysts that significantly lower desorption energy requirements and optimize formulation strategies for mixed amine solutions.

## 6. Conclusions

This review examines organic amine absorption as a primary method for post-combustion CO_2_ capture. Our analysis focuses on the performance and characteristics of two main types of organic amine absorbents: single-component amine absorbents and mixed amine absorbents. Current research aimed at enhancing CO_2_ capture performance emphasizes mixed amines and catalysts, which demonstrate superior CO_2_ capture capabilities and improved absorbent performance. Specifically, adding catalysts to single-component amines can effectively increase the absorption rate and reduce regeneration energy consumption. Meanwhile, multi-component mixed amines generally offer better CO_2_ capture capabilities compared to single-component amines, with varying mixed ratios resulting in different CO_2_ capture effects. Despite these advancements, organic amine absorption methods still face significant challenges. Regeneration energy consumption remains excessively high for both single-component and mixed amine solutions, exceeding acceptable levels for current industrial processes. Additionally, the industrial application of mixed amine solutions requires specialized research for designing and manufacturing efficient absorption/desorption system equipment. Existing desorption devices often suffer from high energy consumption and low efficiency. Therefore, reducing energy consumption during the regeneration process remains a key challenge. Future research should concentrate on developing new catalysts and optimizing mixed amine formulations to further enhance CO_2_ capture performance and reduce energy costs. Addressing these areas will significantly improve the effectiveness and feasibility of organic amine absorption methods for CO_2_ capture. In summary, while organic amine absorption methods are expected to remain a major technology for CO_2_ capture in the foreseeable future, overcoming existing limitations and advancing the technology will necessitate ongoing innovation and research.

## Figures and Tables

**Table 1:** CO_2_ capture characteristics of commonly used organic amine solvents.

Solvent	Structure	Vapor Pressure (kPa)	CO_2_ Absorption Capacity ${(\mathrm{mol}}_{{\mathrm{CO}}_{2}}/{\mathrm{kg}}_{\mathrm{absorbent}})$	Properties	Drawbacks
MEA	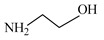	0.064	0.5	Using up to 15–20 wt%	Corrosive Nonselective towards CO_2_ Low capacity for absorption High regeneration energy demand Thermal degradation
AMP	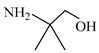	0.1333	0.96	Higher CO_2_ absorption flux The least regeneration energy demand	High reaction heat
PZ	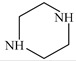	0.279	0.79	Highest reactivity towards CO_2_ High reaction kinetic	-
MDEA	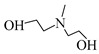	0.001	1.0	Less corrosive Using up to 20–50 wt% Lower degradation rates More economic regeneration	Lower affinity for CO_2_
DGA	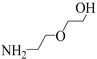	<0.001	0.23–0.35	Using up to 40–60 wt% Low vapor pressure	High reaction heat to CO_2_
DEA	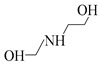	<0.001	0.7	Using up to 35 wt% Less corrosive Lower heat of reaction More economic	Lower reactivity
DIPA	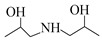	9.3	0.43–0.22	Lower regeneration energy demand Lower corrosivity More degradation resistance	Lower reaction rate than MEA and DEA
N-(2-aminoethyl)ethanolamine (AEEA)	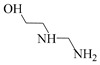	-	2.47	Low cost Lower renewable energy consumption Not easy to degrade	-
DETA	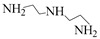	-	5.15	Faster absorption rate Higher CO_2_ absorption load	-

**Table 2:** Improving measures and enhancing performance of organic amine absorption method.

Amine	Measures for Performance Enhancement	Enhanced Performance	References
MEA	SnO_2_/ATP catalyst	Desorption rate and absorption rate increased Regeneration heat duty reduced	[50]
	HZSM-5 catalyst	Absorption rate increased Regeneration energy consumption reduced	[51]
	Metal (Fe, Ni, Mo) supported activated carbon (AC) catalyst	Solvent regeneration capacity increased Desorption rate increased Heat duty reduced	[52]
MDEA	Solid base catalyst	Absorption rate increased	[54]
	DFILs promoter	CO_2_ loading rate increased Regeneration heat duty reduced Absorbent loss rate low	[55]
	MnO catalyst	CO_2_ absorption capacity increased CO_2_ absorption rate improved	[56]
AMP	As a modulator for DA2MP	Solution viscosity reduced Regeneration energy consumption reduced	[58]
	As a modulator for TETA-based SLPCAs	CO_2_ loading capacity increased Absorbent regeneration efficiency improved Formation of gel products avoided	[59]
PZ	As an activator for water-lean amine solvents	Regeneration efficiency improved Energy consumption reduced	[64]
PZ + MDEA	-	Reaction rate increased High regeneration efficiency Lower energy consumption compared to MEA solvent	[80,81]
PZ + TMBPA	1.5 M TMBPA + 1.0 M PZ	Higher reaction rate Higher absorption rate; low cost	[83]
MEA + AMP	-	Reaction rate increased CO_2_ loading increased	[84]
MEA + MDEA + AMP	7 wt% MEA + 3 wt% MDEA + 1 wt% AMP	Desorption rate increased Desorption energy consumption reduce	[85]
AMP + MDEA	Applied in ELM	Enhanced emulsion stability Increased CO_2_ removal rate	[86]
DMBA + DEEA	Biphasic absorbent	Regeneration energy consumption reduced Cost reduced	[92]
DEEA + HMDA	-	Higher CO_2_ solubility Lower absorption heat Smaller packing column volume required Lower equipment cost	[96]
AEEA + DEEA	AEEA as an activator for DEEA aqueous solutio	CO_2_ absorption rate increased	[97]
MEA + DEEA	With a molar ratio of 2.5:2.	CO_2_ cyclic capacity increased CO_2_ absorption rate increased	[98]
-	solid catalyst: HZSM-5,γ-Al_2_O_3_,TiO(OH)_2_	CO_2_ absorption rate increased Regeneration energy consumption reduced	[103,104,105]

## Data Availability

The data are included within the article.

## References

[1] Patel H., Weldekidan H., Mohanty A., Misra M. (2023). Effect of Physicochemical Activation on CO_2_ Adsorption of Activated Porous Carbon Derived from Pine Sawdust. Carbon Capture Sci. Technol..

[2] Patel H., Mohanty A., Misra M. (2024). Post-Combustion CO_2_ Capture Using Biomass Based Activated Porous Carbon: Latest Advances in Synthesis Protocol and Economics. Renew. Sustain. Energy Rev..

[3] Doddapaneni T.R.K.C., Praveenkumar R., Tolvanen H., Rintala J., Konttinen J. (2018). Techno-Economic Evaluation of Integrating Torrefaction with Anaerobic Digestion. Appl. Energy.

[4] Total Increase in Energy-Related CO_2_ Emissions, 1900–2023—Charts–Data & Statistics. https://www.iea.org/data-and-statistics/charts/total-increase-in-energy-related-co2-emissions-1900-2023.

[5] Tapia J.F.D., Lee J.Y., Ooi R.E.H., Foo D.C.Y., Tan R.R. (2018). A Review of Optimization and Decision-Making Models for the Planning of CO_2_ Capture, Utilization and Storage (CCUS) Systems. Sustain. Prod. Consum..

[6] Shove E. (2018). What Is Wrong with Energy Efficiency?. Build. Res. Inf..

[7] Holechek J.L., Geli H.M.E., Sawalhah M.N., Valdez R. (2022). A Global Assessment: Can Renewable Energy Replace Fossil Fuels by 2050?. Sustainability.

[8] Khosroabadi F., Aslani A., Bekhrad K., Zolfaghari Z. (2021). Analysis of Carbon Dioxide Capturing Technologies and Their Technology Developments. Clean. Eng. Technol..

[9] Mondal M.K., Balsora H.K., Varshney P. (2012). Progress and Trends in CO_2_ Capture/Separation Technologies: A Review. Energy.

[10] Meng F., Meng Y., Ju T., Han S., Lin L., Jiang J. (2022). Research Progress of Aqueous Amine Solution for CO_2_ Capture: A Review. Renew. Sustain. Energy Rev..

[11] Omodolor I.S., Otor H.O., Andonegui J.A., Allen B.J., Alba-Rubio A.C. (2020). Dual-Function Materials for CO_2_ Capture and Conversion: A Review. Ind. Eng. Chem. Res..

[12] Liu R.-S., Shi X.-D., Wang C.-T., Gao Y.-Z., Xu S., Hao G.-P., Chen S., Lu A.-H. (2021). Advances in Post-Combustion CO_2_ Capture by Physical Adsorption: From Materials Innovation to Separation Practice. ChemSusChem.

[13] Ochedi F.O., Yu J., Yu H., Liu Y., Hussain A. (2021). Carbon Dioxide Capture Using Liquid Absorption Methods: A Review. Environ. Chem. Lett..

[14] Vega F., Sanna A., Navarrete B., Maroto-Valer M.M., Cortés V.J. (2014). Degradation of Amine-Based Solvents in CO_2_ Capture Process by Chemical Absorption. Greenh. Gases Sci. Technol..

[15] Koronaki I.P., Prentza L., Papaefthimiou V. (2015). Modeling of CO_2_ Capture via Chemical Absorption Processes—An Extensive Literature Review. Renew. Sustain. Energy Rev..

[16] Petrakopoulou F., Tsatsaronis G. (2012). Production of Hydrogen-Rich Fuels for Pre-Combustion Carbon Capture in Power Plants: A Thermodynamic Assessment. Int. J. Hydrogen Energy.

[17] Wang M., Oko E. (2017). Special Issue on Carbon Capture in the Context of Carbon Capture, Utilisation and Storage (CCUS). Int. J. Coal Sci. Technol..

[18] Jansen D., Gazzani M., Manzolini G., van Dijk E., Carbo M. (2015). Pre-Combustion CO_2_ Capture. Int. J. Greenh. Gas Control.

[19] Madejski P., Chmiel K., Subramanian N., Kuś T. (2022). Methods and Techniques for CO_2_ Capture: Review of Potential Solutions and Applications in Modern Energy Technologies. Energies.

[20] Lin H., Zhou M., Ly J., Vu J., Wijmans J.G., Merkel T.C., Jin J., Haldeman A., Wagener E.H., Rue D. (2013). Membrane-Based Oxygen-Enriched Combustion. Ind. Eng. Chem. Res..

[21] Daood S.S., Nimmo W., Edge P., Gibbs B.M. (2012). Deep-Staged, Oxygen Enriched Combustion of Coal. Fuel.

[22] Wang M., Lawal A., Stephenson P., Sidders J., Ramshaw C. (2011). Post-Combustion CO_2_ Capture with Chemical Absorption: A State-of-the-Art Review. Chem. Eng. Res. Des..

[23] Mukherjee A., Okolie J.A., Abdelrasoul A., Niu C., Dalai A.K. (2019). Review of Post-Combustion Carbon Dioxide Capture Technologies Using Activated Carbon. J. Environ. Sci..

[24] Kárászová M., Zach B., Petrusová Z., Červenka V., Bobák M., Šyc M., Izák P. (2020). Post-Combustion Carbon Capture by Membrane Separation, Review. Sep. Purif. Technol..

[25] Olabi A.G., Alami A.H., Ayoub M., Aljaghoub H., Alasad S., Inayat A., Abdelkareem M.A., Chae K.-J., Sayed E.T. (2023). Membrane-Based Carbon Capture: Recent Progress, Challenges, and Their Role in Achieving the Sustainable Development Goals. Chemosphere.

[26] Berstad D., Anantharaman R., Nekså P. (2013). Low-Temperature CO_2_ Capture Technologies – Applications and Potential. Int. J. Refrig..

[27] Ünveren E.E., Monkul B.Ö., Sarıoğlan Ş., Karademir N., Alper E. (2017). Solid Amine Sorbents for CO_2_ Capture by Chemical Adsorption: A Review. Petroleum.

[28] Nie L., Mu Y., Jin J., Chen J., Mi J. (2018). Recent Developments and Consideration Issues in Solid Adsorbents for CO_2_ Capture from Flue Gas. Chin. J. Chem. Eng..

[29] Lee S.-Y., Park S.-J. (2015). A Review on Solid Adsorbents for Carbon Dioxide Capture. J. Ind. Eng. Chem..

[30] Aghel B., Janati S., Wongwises S., Shadloo M.S. (2022). Review on CO_2_ Capture by Blended Amine Solutions. Int. J. Greenh. Gas Control.

[31] Koytsoumpa E.I., Bergins C., Kakaras E. (2018). The CO_2_ Economy: Review of CO_2_ Capture and Reuse Technologies. J. Supercrit. Fluids.

[32] Liang Z., Rongwong W., Liu H., Fu K., Gao H., Cao F., Zhang R., Sema T., Henni A., Sumon K. (2015). Recent Progress and New Developments in Post-Combustion Carbon-Capture Technology with Amine Based Solvents. Int. J. Greenh. Gas Control.

[33] Gatti M., Martelli E., Marechal F., Consonni S. (2014). Review, Modeling, Heat Integration, and Improved Schemes of Rectisol-Based Processes for CO^®^_2_ Capture. Appl. Therm. Eng..

[34] Ebewele E.O., Al-Marzouqi M.H. Regeneration of Solvent for CO_2_ Capture: A Review. Proceedings of the 2021 6th International Conference on Renewable Energy: Generation and Applications (ICREGA).

[35] Li X., Liu J., Jiang W., Gao G., Wu F., Luo C., Zhang L. (2021). Low Energy-Consuming CO_2_ Capture by Phase Change Absorbents of Amine/Alcohol/H_2_O. Sep. Purif. Technol..

[36] Ooi Z.L., Tan P.Y., Tan L.S., Yeap S.P. (2020). Amine-Based Solvent for CO_2_ Absorption and Its Impact on Carbon Steel Corrosion: A Perspective Review. Chin. J. Chem. Eng..

[37] Zhang X., Huang Y., Gao H., Luo X., Liang Z., Tontiwachwuthikul P. (2019). Zeolite Catalyst-Aided Tri-Solvent Blend Amine Regeneration: An Alternative Pathway to Reduce the Energy Consumption in Amine-Based CO_2_ Capture Process. Appl. Energy.

[38] Narku-Tetteh J., Muchan P., Saiwan C., Supap T., Idem R. (2017). Selection of Components for Formulation of Amine Blends for Post Combustion CO_2_ Capture Based on the Side Chain Structure of Primary, Secondary and Tertiary Amines. Chem. Eng. Sci..

[39] Caplow M. Kinetics of Carbamate Formation and Breakdown. https://pubs.acs.org/doi/pdf/10.1021/ja01026a041.

[40] Danckwerts P.V. (1979). The Reaction of CO_2_ with Ethanolamines. Chem. Eng. Sci..

[41] Chen M., Gao H., Sema T., Xiao M., Sun Q., Liang Z. (2022). Study on the Mechanism and Kinetics of Amine with Steric Hindrance Absorbing CO_2_ in Non-Aqueous/Aqueous Solution. Sep. Purif. Technol..

[42] Donaldson T.L., Nguyen Y.N. Carbon Dioxide Reaction Kinetics and Transport in Aqueous Amine Membranes. https://pubs.acs.org/doi/pdf/10.1021/i160075a005.

[43] Hu X., Huang J., He X., Luo Q., Li C., Zhou C., Zhang R. (2022). Analyzing the Potential Benefits of Trio-Amine Systems for Enhancing the CO_2_ Desorption Processes. Fuel.

[44] Waseem M., Al-Marzouqi M., Ghasem N. (2023). A Review of Catalytically Enhanced CO_2_-Rich Amine Solutions Regeneration. J. Environ. Chem. Eng..

[45] Tavakoli A., Rahimi K., Saghandali F., Scott J., Lovell E. (2022). Nanofluid Preparation, Stability and Performance for CO_2_ Absorption and Desorption Enhancement: A Review. J. Environ. Manage..

[46] Asif M., Suleman M., Haq I., Jamal S.A. (2018). Post-Combustion CO_2_ Capture with Chemical Absorption and Hybrid System: Current Status and Challenges. Greenh. Gases Sci. Technol..

[47] Ma’mun S., Jakobsen J.P., Svendsen H.F., Juliussen O. (2006). Experimental and Modeling Study of the Solubility of Carbo-n Dioxide in Aqueous 30 Mass % 2-((2-Aminoethyl)Amino)Ethanol Solution. Ind. Eng. Chem. Res..

[48] McCann N., Phan D., Wang X., Conway W., Burns R., Attalla M., Puxty G., Maeder M. (2009). Kinetics and Mechanism of Carbamate Formation from CO_2_(Aq), Carbonate Species, and Monoethanolamine in Aqueous Solution. J. Phys. Chem. A.

[49] Vinjarapu S.H.B., Neerup R., Larsen A.H., Jørsboe J.K., Villadsen S.N.B., Jensen S., Karlsson J.L., Kappel J., Lassen H., Blinksbjerg P. (2024). Results from Pilot-Scale CO_2_ Capture Testing Using 30 Wt% MEA at a Waste-to-Energy Facility: Optimisation through Parametric Analysis. Appl. Energy.

[50] Tan Z., Zhang S., Zhao F., Zhang R., Tang F., You K., Luo H., Zhang X. (2023). SnO_2_/ATP Catalyst Enabling Energy-Efficient and Green Amine-Based CO_2_ Capture. Chem. Eng. J..

[51] Guo Y., Zhang H., Fu K., Chen X., Qiu M., Fan Y. (2023). Integration of Solid Acid Catalyst and Ceramic Membrane to Boost Amine-Based CO_2_ Desorption. Energy.

[52] Bhatti A.H., Waris M., Kazmi W.W., Bhatti U.H., Min G.H., Park B.C., Baek I.H., Nam S.C. (2022). Metal Impregnated Activated Carbon as Cost-Effective and Scalable Catalysts for Amine-Based CO_2_ Capture. SSRN Electron. J..

[53] Mudhasakul S., Ku H., Douglas P.L. (2013). A Simulation Model of a CO_2_ Absorption Process with Methyldiethanolamine Solvent and Piperazine as an Activator. Int. J. Greenh. Gas Control.

[54] Zhang X., Zhang S., Tang F., Tan Z., Peng Y., Zhao S., Xiang C., Sun H., Zhao F., You K. (2023). Solid Base LDH-Catalyzed Ultrafast and Efficient CO_2_ Absorption into a Tertiary Amine Solution. Chem. Eng. Sci..

[55] Tiwari S.C., Pant K.K., Upadhyayula S. (2023). Energy Efficient CO_2_ Capture in Dual Functionalized Ionic Liquids and N-Methyldiethanolamine Solvent Blend System at Elevated Pressures: Interaction Mechanism and Heat Duties. J. Mol. Liq..

[56] Zhang X., Zhang S., Tan Z., Zhao S., Peng Y., Xiang C., Zhao W., Zhang R. (2023). One-Step Synthesis of Efficient Manganese-Based Oxide Catalyst for Ultra-Rapid CO_2_ Absorption in MDEA Solutions. Chem. Eng. J..

[57] Osagie E., Biliyok C., Di Lorenzo G., Hanak D.P., Manovic V. (2018). Techno-Economic Evaluation of the 2-Amino-2-Methyl-1-Propanol (AMP) Process for CO_2_ Capture from Natural Gas Combined Cycle Power Plant. Int. J. Greenh. Gas Control.

[58] Ma M., Liu Y., Chen Y., Jing G., Lv B., Zhou Z., Zhang S. (2023). Regulatory Mechanism of a Novel Non-Aqueous Absorbent for CO_2_ Capture Using 2-Amino-2-Methyl-1-Propanol: Low Viscosity and Energy Efficient. J. CO_2_ Util..

[59] Tu Z., Han F., Liu C., Wang Y., Wei J., Zhou X. (2023). 2-Amino-2-Methyl-1-Propanol Regulated Triethylenetetramine-Based Nonaqueous Absorbents for Solid-Liquid Phase-Change CO_2_ Capture: Formation of Crystalline Powder Products and Mechanism Analysis. Sep. Purif. Technol..

[60] Rochelle G., Chen E., Freeman S., Van Wagener D., Xu Q., Voice A. (2011). Aqueous Piperazine as the New Standard for CO_2_ Capture Technology. Chem. Eng. J..

[61] Yuan Y., Sherman B., Rochelle G.T. (2017). Effects of Viscosity on CO_2_ Absorption in Aqueous Piperazine/2-Methylpiperazine. Energy Procedia.

[62] Yuan Y., Rochelle G.T. (2019). CO_2_ Absorption Rate and Capacity of Semi-Aqueous Piperazine for CO_2_ Capture. Int. J. Greenh. Gas Control.

[63] Moioli S., Pellegrini L.A. (2015). Physical Properties of PZ Solution Used as a Solvent for CO_2_ Removal. Chem. Eng. Res. Des..

[64] Zhao Y., Zhang Y., Liu Q., Guo X., Cao Y., Xu N., Qi T., Chen Y., Chen S. (2023). Energy-Efficient Carbon Dioxide Capture Using Piperazine (PZ) Activated EMEA+DEEA Water Lean Solvent: Performance and Mechanism. Sep. Purif. Technol..

[65] Mofarahi M., Khojasteh Y., Khaledi H., Farahnak A. (2008). Design of CO_2_ Absorption Plant for Recovery of CO_2_ from Flue Gases of Gas Turbine. Energy.

[66] Salkuyeh Y.K., Mofarahi M. (2012). Comparison of MEA and DGA Performance for CO_2_ Capture under Different Operational Conditions. Int. J. Energy Res..

[67] Guo X.-P., Tomoe Y. (1999). Difference in Corrosion Behavior between Carbon Steel in CO_2_-Saturated MDEA and DGA Solutions. Zair.--Kankyo.

[68] Kierzkowska-Pawlak H. (2010). Carbon Dioxide Removal from Flue Gases by Absorption/Desorption in Aqueous Diethanolamine Solutions. J. Air Waste Manag. Assoc..

[69] Galindo P., Schäffer A., Brechtel K., Unterberger S., Scheffknecht G. (2012). Experimental Research on the Performance of CO_2_-Loaded Solutions of MEA and DEA at Regeneration Conditions. Fuel.

[70] Kim Y.E., Park J.H., Yun S.H., Nam S.C., Jeong S.K., Yoon Y.I. (2014). Carbon Dioxide Absorption Using a Phase Transitional Alkanolamine–Alcohol Mixture. J. Ind. Eng. Chem..

[71] Mavroudi M., Kaldis S.P., Sakellaropoulos G.P. (2003). Reduction of CO_2_ Emissions by a Membrane Contacting Process. Fuel.

[72] Sutar P.N., Jha A., Vaidya P.D., Kenig E.Y. (2012). Secondary Amines for CO_2_ Capture: A Kinetic Investigation Using N-Ethylmonoethanolamine. Chem. Eng. J..

[73] Xu Z., Wang S., Liu J., Chen C. (2012). Solvents with Low Critical Solution Temperature for CO_2_ Capture. Energy Procedia.

[74] Krótki A., Więcław-Solny L., Tatarczuk A., Spietz T., Chwoła T., Dobras S. (2023). A Pilot Study Comparing MEA and AEEA Solvents in Carbon Capture. Int. J. Greenh. Gas Control.

[75] Aso D., Orimoto Y., Higashino M., Taniguchi I., Aoki Y. (2022). Why Does 2-(2-Aminoethylamino)Ethanol Have Superior CO_2_ Separation Performance to Monoethanolamine? A Computational Study. Phys. Chem. Chem. Phys..

[76] Zhang X., Fu K., Liang Z., Rongwong W., Yang Z., Idem R., Tontiwachwuthikul P. (2014). Experimental Studies of Regeneration Heat Duty for CO_2_ Desorption from Diethylenetriamine (DETA) Solution in a Stripper Column Packed with Dixon Ring Random Packing. Fuel.

[77] Ramezani R., Di Felice R. (2021). Kinetics Study of CO_2_ Absorption in Potassium Carbonate Solution Promoted by Diethylenetriamine. Green Energy Environ..

[78] Chakravarty T., Phukan U.K., Weilund R.H. (1985). Reaction of Acid Gases with Mixtures of Amines. Chem. Eng. Prog. U. S..

[79] Muchan P., Saiwan C., Narku-Tetteh J., Idem R., Supap T., Tontiwachwuthikul P. (2017). Screening Tests of Aqueous Alkanolamine Solutions Based on Primary, Secondary, and Tertiary Structure for Blended Aqueous Amine Solution Selection in Post Combustion CO_2_ Capture. Chem. Eng. Sci..

[80] Khan A.A., Halder G.N., Saha A.K. (2017). Experimental Investigation on Efficient Carbon Dioxide Capture Using Piperazine (PZ) Activated Aqueous Methyldiethanolamine (MDEA) Solution in a Packed Column. Int. J. Greenh. Gas Control.

[81] Hosseini-Ardali S.M., Hazrati-Kalbibaki M., Fattahi M., Lezsovits F. (2020). Multi-Objective Optimization of Post Combustion CO_2_ Capture Using Methyldiethanolamine (MDEA) and Piperazine (PZ) Bi-Solvent. Energy.

[82] Oh S.-Y., Yun S., Kim J.-K. (2018). Process Integration and Design for Maximizing Energy Efficiency of a Coal-Fired Power Plant Integrated with Amine-Based CO_2_ Capture Process. Appl. Energy.

[83] Aronu U.E., Hoff K.A., Svendsen H.F. (2011). CO_2_ Capture Solvent Selection by Combined Absorption–Desorption Analysis. Chem. Eng. Res. Des..

[84] Choi W.-J., Seo J.-B., Jang S.-Y., Jung J.-H., Oh K.-J. (2009). Removal Characteristics of CO_2_ Using Aqueous MEA/AMP Solutions in the Absorption and Regeneration Process. J. Environ. Sci..

[85] Najib S.B.M., Kamaruddin K.S.N., Rashid N.M., Ibrahim N., Sokri M.N.M., Zaini N., Nordin N. (2022). The Effect of MDEA/AMP and Span-80 in Water-in-Oil (W/O) Emulsion for Carbon Dioxide Absorption. J. Appl. Membr. Sci. Technol..

[86] Liu Y., Fan W., Wang K., Wang J. (2016). Studies of CO_2_ Absorption/Regeneration Performances of Novel Aqueous Monothanlamine (MEA)-Based Solutions. J. Clean. Prod..

[87] Wang L., An S., Yu S., Zhang S., Zhang Y., Li M., Li Q. (2017). Mass Transfer Characteristics of CO_2_ Absorption into a Phase-Change Solvent in a Wetted-Wall Column. Int. J. Greenh. Gas Control.

[88] Raksajati A., Ho M.T., Wiley D.E. (2014). Reducing The Cost of CO_2_ Capture From Flue Gases Using Phase-Change Solvent Absorption. Energy Procedia.

[89] Pinto D.D.D., Zaidy S.A.H., Hartono A., Svendsen H.F. (2014). Evaluation of a Phase Change Solvent for CO_2_ Capture: Absorption and Desorption Tests. Int. J. Greenh. Gas Control.

[90] Shen Y., Jiang C., Zhang S., Chen J., Wang L., Chen J. (2018). Biphasic Solvent for CO_2_ Capture: Amine Property-Performance and Heat Duty Relationship. Appl. Energy.

[91] Gautam A., Mondal M.K. (2023). Review of Recent Trends and Various Techniques for CO_2_ Capture: Special Emphasis on Biphasic Amine Solvents. Fuel.

[92] Wang L., An S., Li Q., Yu S., Wu S. (2017). Phase Change Behavior and Kinetics of CO_2_ Absorption into DMBA/DEEA Solution in a Wetted-Wall Column. Chem. Eng. J..

[93] Singh P., van Swaaij W.P.M., Wim Brilman D.W.F. (2011). Kinetics Study of Carbon Dioxide Absorption in Aqueous Solutions of 1,6-Hexamethyldiamine (HMDA) and 1,6-Hexamethyldiamine, N,N′ Di-Methyl (HMDA, N,N′). Chem. Eng. Sci..

[94] Sutar P.N., Vaidya P.D., Kenig E.Y. (2013). Activated DEEA Solutions for CO_2_ Capture—A Study of Equilibrium and Kinetic Characteristics. Chem. Eng. Sci..

[95] Kim S., Kim M., Kim J. (2019). Techno-Economic Evaluation and Comparative Analysis of the CO_2_ Separation Processes Using Different Piperazine-Mixed Amine Absorbents. J. Chem. Eng. Jpn..

[96] Bai L., Zhao D., Zhong X., Dong S., Liu H. (2023). Comprehensive Technical Analysis of CO_2_ Absorption into a Promising Blended Amine of DEEA-HMDA. Chem. Eng. Sci..

[97] Kierzkowska-Pawlak H. (2015). Kinetics of CO_2_ Absorption in Aqueous *N*,*N*-Diethylethanolamine and Its Blend with *N*-(2-Aminoethyl)Ethanolamine Using a Stirred Cell Reactor. Int. J. Greenh. Gas Control.

[98] Luo X., Liu S., Gao H., Liao H., Tontiwachwuthikul P., Liang Z. (2016). An Improved Fast Screening Method for Single and Blended Amine-Based Solvents for Post-Combustion CO_2_ Capture. Sep. Purif. Technol..

[99] de Meyer F., Bignaud C. (2022). The Use of Catalysis for Faster CO_2_ Absorption and Energy-Efficient Solvent Regeneration: An Industry-Focused Critical Review. Chem. Eng. J..

[100] Alivand M.S., Mazaheri O., Wu Y., Stevens G.W., Scholes C.A., Mumford K.A. (2020). Catalytic Solvent Regeneration for Energy-Efficient CO_2_ Capture. ACS Sustain. Chem. Eng..

[101] Zhang X., Hong J., Liu H., Luo X., Olson W., Tontiwachwuthikul P., Liang Z. (2018). SO_4_^2−^/ZrO_2_ Supported on γ-Al_2_O_3_ a-s a Catalyst for CO_2_ Desorption from CO_2_-Loaded Monoethanolamine Solutions. AIChE J..

[102] Ali Saleh Bairq Z., Gao H., Huang Y., Zhang H., Liang Z. (2019). Enhancing CO_2_ Desorption Performance in Rich MEA Solution by Addition of SO_4_^2−^/ZrO_2_/SiO_2_ Bifunctional Catalyst. Appl. Energy.

[103] Shi H., Naami A., Idem R., Tontiwachwuthikul P. (2014). Catalytic and Non Catalytic Solvent Regeneration during Absorp-tion-Based CO_2_ Capture with Single and Blended Reactive Amine Solvents. Int. J. Greenh. Gas Control.

[104] Bhatti U.H., Shah A.K., Hussain A., Khan H.A., Park C.Y., Nam S.C., Baek I.H. (2020). Catalytic Activity of Facilely Synt-hesized Mesoporous HZSM-5 Catalysts for Optimizing the CO_2_ Desorption Rate from CO_2_-Rich Amine Solutions. Chem. Eng. J..

[105] Lai Q., Toan S., Assiri M.A., Cheng H., Russell A.G., Adidharma H., Radosz M., Fan M. (2018). Catalyst-TiO(OH)_2_ Coul-d Drastically Reduce the Energy Consumption of CO_2_ Capture. Nat. Commun..

[106] Bae T.-H., R. Hudson M., A. Mason J., L. Queen W., J. Dutton J., Sumida K., J. Micklash K., S. Kaye S., M. Brown C., R. Long J. (2013). Evaluation of Cation -Exchanged Zeolite Adsorbents for Post- Combustion Carbon Dioxide Capture. Energy Environ. Sci..

